# An Overview of Growth Factors as the Potential Link between Psoriasis and Metabolic Syndrome

**DOI:** 10.3390/jcm13010109

**Published:** 2023-12-24

**Authors:** Mateusz Matwiejuk, Hanna Myśliwiec, Adrian Chabowski, Iwona Flisiak

**Affiliations:** 1 Department of Dermatology and Venereology, Medical University of Bialystok, 15-089 Bialystok, Poland; 2 Department of Physiology, Medical University of Bialystok, 15-089 Bialystok, Poland

**Keywords:** bFGF, HGF, NGF b, SCF, PDGF-BB, M-CSF, skin, skin diseases, psoriasis, metabolic syndrome

## Abstract

Psoriasis is a chronic, complex, and immunologically mediated systemic disease that not only affects the skin, but also the joints and nails. It may coexist with various other disorders, such as depression, psoriatic arthritis, cardiovascular diseases, diabetes mellitus, and metabolic syndrome. In particular, the potential link between psoriasis and metabolic syndrome is an issue worthy of attention. The dysregulation of growth factors could potentially contribute to the disturbances of keratinocyte proliferation, inflammation, and itch severity. However, the pathophysiology of psoriasis and its comorbidities, such as metabolic syndrome, remains incompletely elucidated. Growth factors and their abnormal metabolism may be a potential link connecting these conditions. Overall, the objective of this review is to analyze the role of growth factor disturbances in both psoriasis and metabolic syndrome.

## 1. Introduction

Psoriasis is an immune-caused, chronic, and relapsing inflammatory disease which is widespread across the world [1]. The prevalence of psoriasis in adults ranges between 0.51 and 11.43% while, in children, it has been estimated to be between 0% and 1.37%. More precisely, the prevalence of psoriasis is at a high level in countries in Western Europe (1.92% of citizens) and Central Europe (1.83% of residents), as well as in North America (1.50% of inhabitants) [2]. Psoriasis can be divided into different sub-types, including plaque, pustular, and erythrodermic psoriasis [3]. Upon histological examination, psoriatic skin tissue is characterized by elongated rete pegs, thickening (acanthosis) of the epidermis, incomplete keratinocyte differentiation, abnormal proliferation, and parakeratosis (keratinocytes aberrantly retaining intact nuclei) [4].

Psoriasis is often linked with other comorbidities, such as diabetes mellitus, obesity, hypertension, dyslipidemia, and non-alcoholic fatty liver disease (NAFLD) [5]. Among the components of metabolic syndrome, the following elements are most commonly associated with psoriasis: obesity, hypertension, atherogenic dyslipidemia, high triglyceride levels, and insulin resistance [6]. It is known that various growth factors may have different functions in maintaining proper cellular proliferation, differentiation, apoptosis, and homeostasis [7]. Having spoken of psoriatic modalities, at present there is no cure for psoriasis, but many effective treatment options are available that can help to manage the symptoms and improve the quality of life of patients. Topical therapy is the most common treatment for mild to moderate psoriasis, and can be used alone or in combination with other treatments. Systemic therapy, which includes medications that are taken by mouth or injection, is used for more severe psoriasis or when topical therapy is not effective [8]. The development of biological agents within the past two decades has dramatically boosted the treatment of psoriasis and psoriatic arthritis. At present, 11 Food and Drug Administration (FDA)-approved biologic options are available for psoriasis modalities: The TNF-alpha inhibitors adalimumab, infliximab, and certolizumab; the Il-17 inhibitors secukinumab, brodalumab, ixekizumab, and bimekizumab; the Il-23 inhibitors guselkumab, tildrakizumab, and risankizumab; and the Il-12/23 inhibitor ustekinumab [9].

A growth factor is a naturally occurring substance, typically a secreted protein or a steroid hormone, that stimulates cell proliferation, wound healing, and occasionally cellular differentiation. Growth factors are essential for regulating various cellular processes and act as signaling molecules, binding to specific receptors on the surface of target cells. These receptors activate intracellular signaling pathways that ultimately lead to changes in gene expression and cellular behavior. Growth factors play a complex and significant role in inflammation and are crucial for cell recruitment, angiogenesis, and extracellular matrix remodeling. Growth factors can influence the production and release of cytokines and chemokines, maintaining or resolving inflammation.

In recent years, studies have mainly focused on different aspects of the potential role of specific growth factors in the pathogenesis of many conditions, including psoriasis and metabolic syndrome. While the precise mechanism remains incompletely elucidated, the interconnection between these two conditions through growth factors remains an area of ongoing investigation. The present review aims to evaluate the potential association between growth factors and the common occurrence of psoriasis and metabolic syndrome. We focused our study on GFs which could be a significant link between psoriasis and metabolic syndrome, but this information is not widely known. We also include some important facts about GFs that are already well-established in the pathogenesis of these two conditions.

## 2. Materials and Methods

This systematic review was conducted in accordance with the 2020 updated Preferred Reporting Items for Systematic Reviews and Meta-Analyses (PRISMA) guidelines [10,11]. The review protocol was submitted to the Protocols database.

A medical literature search of PubMed (1985–present), conducted in the spring of 2023, was performed using appropriate terms without date limitations. The main object of the research was to identify the role of growth factors as a potential bridge between metabolic syndrome and psoriasis. Medical subject headline terms included “b-FGF and skin”, “b-FGF and skin diseases”, “b-FGF and psoriasis”, “b-FGF and metabolic syndrome”, “HGF and skin”, “HGF and skin diseases”, “HGF and psoriasis”, “HGF and metabolic syndrome”, “NGFb and skin”, “NGFb and skin diseases”, “NGF and psoriasis”, “NGFb and metabolic syndrome”, “SCF and skin”, “SCF and skin diseases”, “SCF and psoriasis”, “SCF and metabolic syndrome”, “PDGF-BB and skin”, “PDGF-BB and skin diseases”, “PDGF-BB and psoriasis”, “PDGF-BB and metabolic syndrome”, “M-CSF and skin”, “M-CSF and skin diseases”, “M-CSF and psoriasis”, “M-CSF and metabolic syndrome”, “VEGF and skin”, “VEGF and skin diseases”, “VEGF and psoriasis”, “VEGF and metabolic syndrome”, “TGF-α and skin”, “TGF-α and skin diseases”, “TGF-α and psoriasis”, “TGF-α and metabolic syndrome”, “TGF-β and skin”, “TGF-β and skin diseases”, “TGF-β and psoriasis”, “TGF-β and metabolic syndrome”, “EGF and skin”, “EGF and skin diseases”, “EGF and psoriasis”, “EGF and metabolic syndrome”, “IGF and skin”, “IGF and skin diseases”, “IGF and psoriasis”, and “IGF and metabolic syndrome”.

Non-English publications, papers with low clinical significance, papers written in a language other than English, and duplicated publications were excluded from the analysis. Originally, human and animal studies were included in this systematic review. The results of the search strings were combined together, and duplicates were removed. Afterwards, the titles and abstracts of the searched studies were independently screened by two reviewers (M.M. and H.M.) in order to identify relevant articles that addressed the review subject. Disagreements between reviewers were resolved by a third reviewer (A.C.). Finally, the selected eligible articles were fully reviewed.

## 3. Results

**Figure 1:**
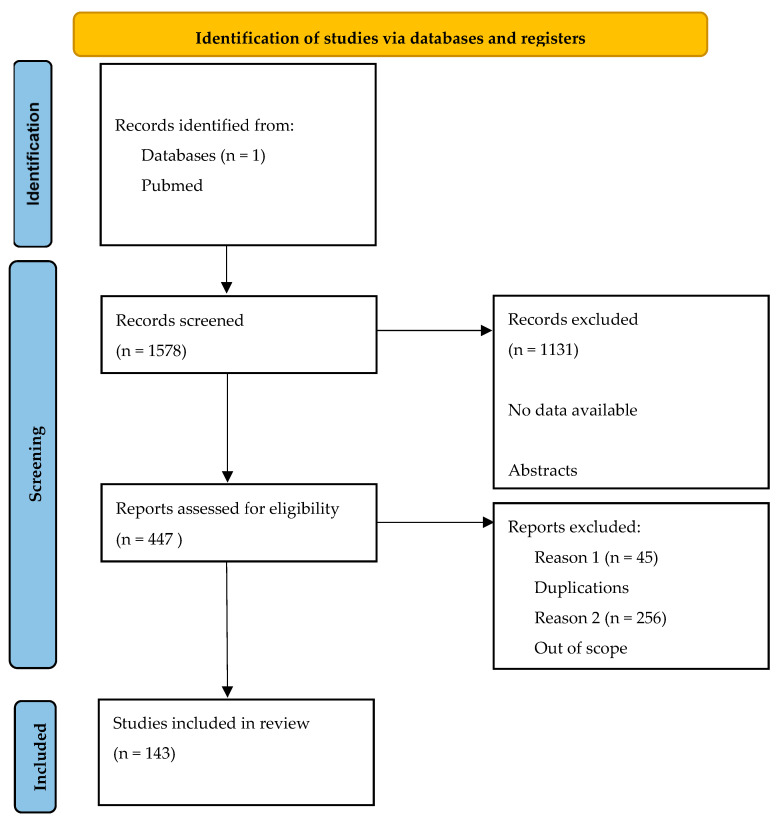
The search process.

According to Figure 1, the search resulted in the retrieval of 5335 records, of which 1578 were screened for relevance and 143 were ultimately included in the qualitative synthesis.

## 4. Discussion

**Figure 2:**
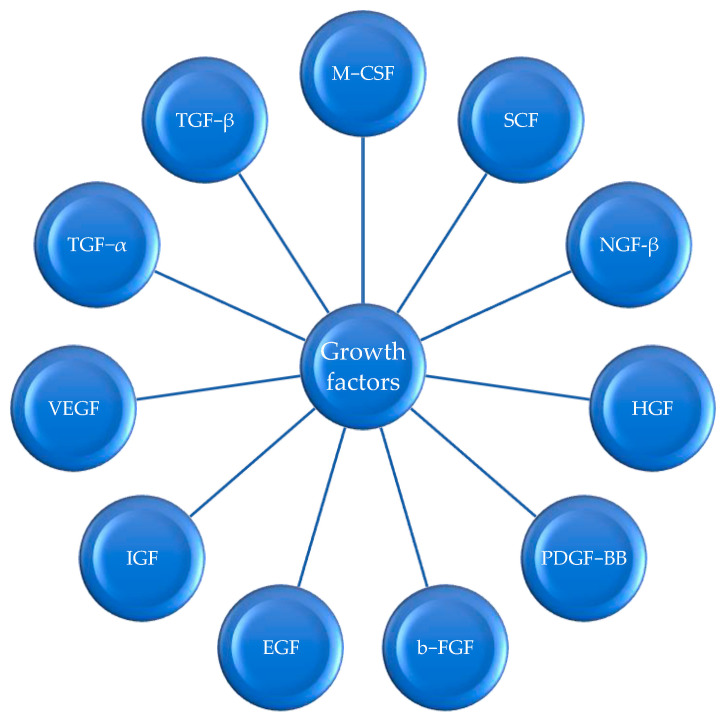
Examples of growth factors.

**Figure 3:**
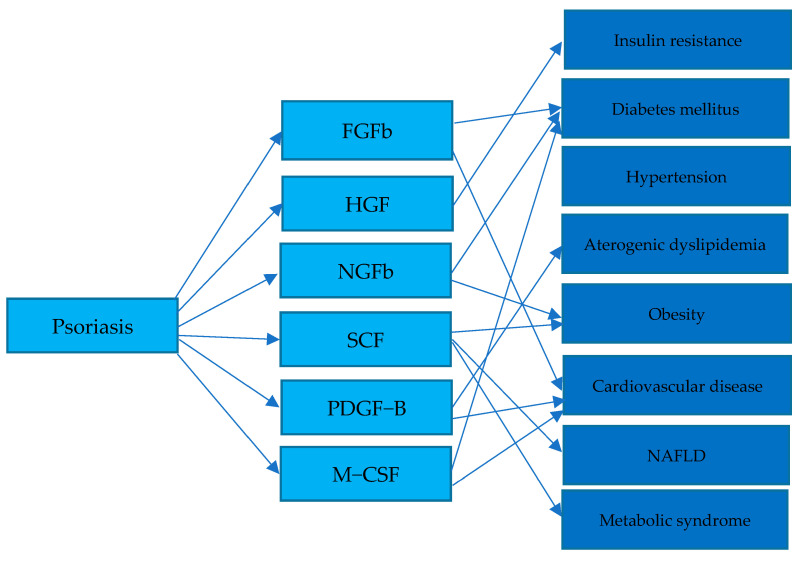
The potential mediating roles of chemokines between psoriasis and its comorbidities.

Growth factors are a class of signaling molecules that are essential for regulating a variety of cellular processes, including proliferation, differentiation, angiogenesis, survival, inflammation, and tissue repair or fibrosis. They are typically secreted proteins or steroid hormones, which exert their effects by binding to specific receptors on the surface of target cells. In this paper, we focus on several growth factors which are plausibly implicated in the pathogenesis of both psoriasis and metabolic syndrome, suggesting a potential link between these two conditions. There is a general group of growth factors, which includes basic fibroblast growth factor (b-FGF), platelet-derived growth factor (PDGF-BB), hepatocyte growth factor (HGF), nerve growth factor beta (NGF-β), stem cell factor (SCF), macrophage colony-stimulating factor (M-CSF), vascular endothelial growth factor (VEGF), transforming growth factor alpha (TGF-α), transforming growth factor β (TGF-β), epidermal growth factor (EGF), and insulin-like growth factor (IGF). Some of these growth factors are shown below in Figure 2. Furthermore, it is hypothesized that the pathophysiology of psoriasis and its comorbidities, such as metabolic syndrome, is linked through growth factors and their abnormal metabolism, as shown in Figure 3.

### 4.1. FGF and Skin

Basic fibroblast growth factor (b-FGF), also known as fibroblast growth factor 2 or FGF-β, is produced by endothelial cells and fibroblasts and targets fibroblasts and endothelial cells. Like other FGF family members, b-FGF plays important roles in cell growth, tissue repair, and angiogenesis. Takehara [12] revealed, in his study, that b-FGF is the most potent growth stimulator factor among the other ones and that b-FGF injection caused slight edematous granulated tissue formation [12]; see Table 1.

Makino et al. [13] reported that b-FGF increased the number of human dermal fibroblasts (HDFs). This b-FGF-induced proliferation was suppressed by the Jun N-terminal kinase (JNK) inhibitor SP600125 and the mitogen-activated protein kinase (MEK) inhibitors PD98059 and U0126. Moreover, b-FGF increased the phosphorylation levels of extracellular signal-regulated kinase 1/2 (ERK1/2) and Jun N-terminal kinase 1 (JNK1). Treatment with ERK1, ERK2, or JNK1 siRNA significantly inhibited b-FGF-induced proliferation. The authors showed that the ERK1/2 and JNK pathways play an essential role in the b-FGF-mediated effect in HDFs. Additionally, it was also concluded that controlling ERK1/2 and/or JNK signaling might provide a new therapeutic means for the treatment of chronic skin ulcers [13]; see Table 1.

### 4.2. FGF in Skin Diseases

Song et al. [14] showed that treatment with b-FGF promotes cell migration, which is an important factor in wound healing processes. Moreover, b-FGF treatment boosted the activity of c-Jun N-terminal kinase (JNK), a key regulator of fibroblast cell migration [14]. In addition, Nakamizo et al. [15] showed that b-FGF increased the expression of Ki67 in keratinocytes following mechanical scratching. These findings, consistent with prior studies [15], indicated that b-FGF boosts the proliferation of keratinocytes. This proliferation process aids in the restoration of the skin barrier after disruption due to scratching in mice. In conclusion, b-FGF stimulated the proliferation of normal human epidermal keratinocytes (NHEK) [15]; see Table 2.

Qu et al. [16] reported the results from their in vitro studies and confirmed that implementing keratinocyte growth factor (KGF) and b-FGF into mouse skin resulted in an acceleration of cell migration and an increase in cellular proliferation rates. After the process of transplantation of the biomaterial onto an excisional wound healing model, the dual growth factor group compared to the single growth factor groups and empty control group, showed better-organized epidermal regeneration in the wound’s vascular networks. In the results, this experimental group showed mature epidermal coverage. In summary, these outcomes fulfill clinical needs for wound care [16]; see Table 2.

Interestingly, Wu et al. [17] noted, in their study, that b-FGF is ineffective in the setting of ischemia, suggesting that tissue hypoxia may inhibit certain growth factors ineffectively. Under aerobic conditions, b-FGF achieves proper efficacy, which indicates the profound modulation of growth factor function by oxygen [17].

Richard et al. [18] administered topical b-FGF treatment to patients suffering from diabetic neuropathic foot ulcers; however, their outcomes did not yield satisfactory results. The outcome of this study indicates that the role of topical b-FGF in the healing of diabetic ulcers is limited [18]; see Table 2.

### 4.3. FGF in Psoriasis

Watanabe et al. [19] proved that the serum b-FGF level was lower in psoriatic arthritis (PsA) and generalized pustular psoriasis (GPP) patients in comparison to healthy people. Interestingly, levels of b-FGF in the sera of PsA and GPP patients normalized to levels similar to those observed in healthy controls following systemic treatment. Indeed, the serum b-FGF level was positively correlated with the psoriasis area and severity index (PASI), as well as the count of circulating eosinophils in GPP patients [19]; see Table 3.

Sharpe et al. [20] noticed that human endothelial cells and keratinocytes driven by b-FGF alone can be inhibited by oral administration of cyclosporine A. Thus, b-FGF may be an important signal driving both keratinocyte proliferation and angiogenesis in psoriasis. Keratinocytes and endothelial cells produce b-FGF, which is mitogenic for both cell types. Displacement of b-FGF by physical stimuli, such as rubbing or scratching, could explain the Koebnerization process in diseases such as psoriasis [20]; see Table 3.

Oppositely, Przepiera-Bedzak et al. [21] observed no significant correlations between clinical presentation and b-FGF and acidic fibroblast growth factor (a-FGF) levels in patients suffering from PsA. Moreover, in patients dealing with synovitis, acne, pustulosis, hyperostosis, and osteitis syndrome (SAPHO), no relevant correlation was identified between the aforementioned cytokine levels and the clinical presentation [21]; see Table 3.

### 4.4. FGF in Metabolic Syndrome

Ivannikova et al. [22] provided evidence of a significant two-fold increase in b-FGF in patients dealing with coronary heart disease (CHD), type 2 diabetes mellitus (T2DM), and acute coronary syndrome (ACS) in comparison to healthy people [22]; see Table 4.

### 4.5. HGF in the Skin

Qin et al. [23] discovered that hepatocyte growth factor (HGF) is mainly expressed in human skin dermal fibroblasts. It plays a pivotal role in collagen production and shows a significant increase within aged human skin in vivo. Furthermore, the decreased size of fibroblasts, which is one of the prominent features of aged skin fibroblasts in vivo, is responsible for the age-related elevation of HGF expression. Raised transcription factor (c-Jun) and impaired transforming growth factor β (TGF-β) signaling led to cell size-dependent up-regulation of HGF expression. Interestingly, the restoration process of fibroblast size was related to elevated c-Jun expression and impaired TGF-β signaling and, thus, reversed the elevated HGF expression. In conclusion, the authors showed that the application of retinoid (ROL), which improves aged human skin, significantly reduced elevated HGF mRNA expression in aged human skin in vivo [23]; see Table 5.

Gron et al. [24] demonstrated that keratinocytes boost HGF and keratinocyte growth factor (KGF) output in cultured oral and skin fibroblasts. The quantity of KGF and HGF production is dependent on the type of fibroblast. Their main finding was that the constitutive level of KGF and HGF in periodontal fibroblasts was elevated in comparison to that in buccal and skin fibroblasts. In summary, this elevated growth factor production may lead to the migration and proliferation of junctional epithelium, thereby influencing the development of periodontal disease [24].

Recio et al. [25] demonstrated, in their study, that the enhanced activity of HGF/c-MET- (mesenchymal–epithelial transition factor) may increase their invasive capacity, boost the proliferation of melanoma cells, and protect melanoma cells from apoptosis [25]; see Table 5.

Moreover, Zeng et al. [26] showed that a high level of HGF may inhibit apoptosis induced by anoikis in head and neck squamous cell carcinoma (SCC) [26]; see Table 5.

### 4.6. HGF in Skin Diseases

Nicu et al. [27] noted, in their study, that dermal white adipose tissue (dWAT) controls pigmentation and human hair growth through HGF secretion. In this case, dWAT and HGF can be considered important novel molecules and potential cellular targets for therapeutic intervention in human hair growth and pigmentation disorders [27]; see Table 6.

Bevan et al. [28] reported that hepatocyte growth factor/scatter factor (HGF/SF) affects and sustains all essential cellular processes that are in charge of wound repair, which point to possible therapeutic means for the treatment of chronic skin wounds. In particular, HGF/SF improved wound repair in homozygous diabetic db/db mice, first through the attraction of mast cells, neutrophils, and monocytes to the wound; then, by enhancing keratinocyte proliferation and migration; and, finally, by promoting the movement of endothelial cells to the injured site. As a consequence of this outcome, wound angiogenesis, the formation of granulation tissue, and the re-epithelialization process are all augmented [28]; see Table 6.

According to Otsuka et al. [29], melanoma cells possess the ability to express both cellular mesenchymal–epithelial transition factor (c-MET) and HGF [29]; see Table 6.

### 4.7. HGF in Psoriasis

Meng et al. [30] reported the therapeutic effect of HGF over-expressed dental pulp stem cells (DPSCs) on imiquimod-induced psoriasis. This study indicated HGF over-expression enhanced the immunoregulation abilities of DPSCs by down-regulating T-helper 1 (Th1) and Th17 cells while up-regulating regulatory T (Treg) cells. In psoriatic skin lesions, psoriasis-like erythema, scaling, and thickening were alleviated. Furthermore, the expression levels of cytokeratin 6 (CK6), cytokeratin 17 (CK17) interferon-gamma (IFN-γ), tumor necrosis factor-α (TNF-a), interleukin (IL)-17A, IFN-γ, retinoic acid-related orphan receptor-γt (RORγt), IL-17A, IL-17F, and IL-23 were down-regulated with DPSCs and HGF-DPSCs treatment in psoriatic skin lesions. As a consequence, HGF over-expression enhanced treatment effect of DPSCs on psoriasis mainly by reducing inflammatory responses. These findings may offer a novel approach for the immunoregulation-based treatment of psoriasis [30]; see Table 7.

Takahashi et al. [31] revealed, in their study, that the significant induction of TNF-alpha increased HGF levels, although an increase of this growth factor resulted in minimal variation between psoriatic skin and normal skin [31]; see Table 7.

### 4.8. HGF in Metabolic Syndrome

Balaban et al. [32] showed that serum HGF levels were strongly associated with insulin resistance (IR) and all components of metabolic syndrome (MS). They showed that serum levels of HGF were also elevated in non-alcoholic fatty liver disease (NASH). IR and MS are features of NASH, and HGF might be the potential link between hepatocytes and adipocytes [32]; see Table 8.

Hiratsuka et al. [33] indicated, in their study, that serum HGF levels are strongly associated with metabolic syndrome; however, contrary to the previous study, they were independent of liver function. Furthermore, HGF levels were significantly related to high-density lipoprotein (HDL; *p* < 0.05, inversely), waist circumference (*p* < 0.001), and liver enzymes (*p* < 0.001). Interestingly, HGF levels were higher (*p* < 0.05) in correlation with the number of components of MS [33]; see Table 8.

Faber et al. [34] reported that visceral adipose tissue (VAT)—but not subcutaneous adipose tissue—is associated with circulating level of HGF, regardless of body mass index (BMI). Furthermore, as in the previous studies, MS was associated with elevated levels of HGF [34]; see Table 8.

Sakaue et al. [35] revealed a significant inverse association between physical activity (PA) changes and HGF levels. Their data indicated that an improvement in PA levels is associated with reduced HGF levels and cardiovascular disease (CVD) development. An increased PA group showed reduced CVD development compared to the stable low PA group (*p* = 0.012), and the HGF levels in the increased PA group were significantly lower than those in the stable low PA group (*p* = 0.038) [35]; see Table 8.

Tsukagawa et al. [36] also indicated that raised serum levels of HGF are significantly linked with the development of insulin resistance (IR). In patients with IR, serum HGF levels were higher (0.26 ± 0.10 ng/mL, n = 259) than in those without IR (0.22 ± 0.09 ng/mL, n = 1090). In addition, a significant (*p* < 0.05) relative risk [1.75 (95%CI: 1.01–3.12)] for the progression of IR was observed for the highest concentration of HGF (≥0.30 ng/mL) in comparison to the lowest groups (<0.15 ng/mL) [36]; see Table 8.

Sanchez-Escinales et al. [37] indicated that muscle expression of HGF prevents obesity-mediated muscle insulin resistance and boosts glucose tolerance in mice. Importantly, obese transgenic mice (SK-HGF) showed improved whole-body glucose tolerance independently of alterations in plasma triglyceride levels, body weight, or compared to control mice. In particular, muscle HGF levels exhibited a three-fold increase in transgenic mice (SK-HGF) compared to the control mice [37]; see Table 8.

Motone et al. [38] suggested, in their study, that C/A polymorphism in intron 13 of the HGF gene is connected with susceptibility to essential hypertension in lean or female subjects, but not in obese or male subjects. Moreover, serum HGF levels are scaled up in response to hypertensive organ damage, leading to the conclusion that alterations of blood pressure may be affected by HGF gene polymorphisms through serum HGF [38]; see Table 8.

### 4.9. NGF-β in Skin Diseases

Sun et al. [39] showed that collagen membranes filled with collagen-targeting human nerve growth factor-beta (NGF-β) effectively enhance ulcer healing. NGF-β promotes the re-epithelialization of dermal ulcer wounds and the formation of capillary lumens in the area of the newly created tissue [39]; see Table 9.

Sari et al. [40] indicated that NGF-β could serve as a marker of pruritus in the elderly, with associated inflammation of the skin and impaired skin barrier. Furthermore, a noteworthy finding was the significant correlation between lower skin pH values (indicating a better skin condition) and reduced NGF-β levels (*p* = 0.035) [40]; see Table 9.

Furthermore, Solinski et al. [41] also revealed that patients dealing with chronic itch were also diagnosed with an elevated level of NGF-β due to inflammatory conditions [41]; see Table 9.

Peng et al. [42] observed increased expression of NGF-β on skin mast cells (MCs). Interestingly, the level of NGF-β was correlated with tryptase levels, which led to the conclusion that there is a potential link between MC load and blood levels of NGF-β. Moreover, the influx of CD117+ progenitor cells from the blood was enhanced toward the NGF-β gradient in both mastocytoses [42]; see Table 9.

### 4.10. NGF-β in Psoriasis

Baerveldt et al. [43] demonstrated that the mRNA expression of psoriasis-related markers such as NGF-β, according to Raychaudhuri [44], was significantly decreased after 4 weeks of ustekinumab injection. It is commonly known that K252a—a selective inhibitor of the tyrosine protein kinase activity, which is an NGF-β receptor antagonist—improves symptoms of psoriasis [43]; see Table 10.

### 4.11. NGF-β in Metabolic Syndrome

Molnar et al. [45] revealed that, in obese and post-menopausal women, higher NGF-β levels were found when compared to healthy women. Moreover, NGF-β levels can play a role in the reduction of serum-free thyroxine (FT_4_) levels in post-menopausal and obese women. On the other hand, decreased levels of FT_4_ have been associated with increased NGF-β in obese women [45]; see Table 11.

Sisman et al. [46] found that a reduced NGF-β level was linked with increased apoptosis and testicular damage in diabetic rats. The testis NGF-β level might be a possible novel rate for assessing diabetes-induced testicular damage [46]; see Table 11.

Ueyama et al. [47] proved that reduced production of NGF-β from vascular smooth muscle cells (VSMCs) may be responsible for the hypotrophy of sympathetic nerve cells in genetically hypertensive rats. In contrast, the secretion of NGF-β per cell was higher in normotensive (NT) rats [47]; see Table 11.

Selavaraju et al. [48] revealed that the salivary amount of NGF-β was significantly elevated in obese children compared to that in normal-weight children. Furthermore, NGF is also positively associated with salivary insulin, blood pressure, obesity, and anthropometric measures. In summary, these findings suggest that NGF-β could serve as a predictive marker for obesity-related complications in children [48]; see Table 11.

### 4.12. SCF in the Skin

Franke et al. [49] showed that the cAMP response element binding protein (CREB) works as a stimulus-inducible transcription factor (TF) that initiates multiple cellular changes in response to activation. Further, CREB is quickly phosphorylated on serine-133 upon stem cell factor (SCF)-mediated KIT dimerization. SCF efficiently induces immediate early genes (IEGs) in skin mast cells (skMCs; *FOS*, *JUNB*, and *NR4A2*). The TF CREB is an essential crucial intermediary in SCF-triggered KIT activation of human skMCs [49]; see Table 12. 

### 4.13. SCF in Skin Diseases

Yamanaka-Takaichi et al. [50] showed that SCF expression within seborrheic keratosis is significantly elevated in comparison to the marginal lesion. Besides, cannabinoid receptor type 1 (CB1), which is a cognitive regulator of SCF expression, was down-regulated in SK lesions. This result highlights that the CB1–SCF–MC (mast cell) interaction is a novel mechanism of SK development. Additionally, these findings may serve as a basis for the development of novel treatments. SCF influences MC differentiation, proliferation, survival, and migration through the c-Kit receptor. Indeed, SCF may also potentially play a crucial role in SK pathogenesis. Nonetheless, the exact mechanism by which SCF triggers these effects remains to be fully understood [50]; see Table 13.

### 4.14. SCF in Psoriasis

Cho et al. [51] noted an increase in dermal mast cells caused by the expression of epidermal SCF. This phenomenon was described in imiquimod-induced psoriatic dermatitis in mice. Likewise, in mouse epidermal keratinocytes, SCF was highly expressed in HaCaTs following stimulation with IL-17 [51]; see Table 14. 

Yamamoto et al. [52] also found that serum SCF levels were elevated in patients with psoriasis vulgaris in comparison to healthy people. Apart from this, serum SCF did not show a correlation with PASI ratio, which describes the severity of the disease. Interestingly, SCF takes part in the ramped-up number of mast cells in the papillary dermis of psoriasis, which may be responsible for the pruritus linked with psoriasis [52]; see Table 14. 

### 4.15. SCF in Metabolic Syndrome

Wang et al. [53] reported that SCF expression in the epidermis is lower in mice with delayed wound closure intended to mimic alcoholism, old age, and obesity. In particular, SCF deficiency in keratinocytes disturbed the migration of both normal fibroblasts and keratinocytes. Additionally, within 24–48 h post-wounding, this deficiency may also lead to a reduction in early neutrophil recruitment [53].

Jialal et al. [54] showed, in their study, that there is a decrease in plasma concentration of stem cell factor (SCF) in obese males. In addition, SCF and SCF-soluble receptors were lowered with a functional deficiency of vascular endothelial growth factor (VEGF) [54]; see Table 15.

He et al. [55] revealed that the SCF^JFK^–ING5 (Inhibitor of growth protein 5) axis interacts with adenosine 5′-monophosphate (AMP)-activated protein kinase (AMPK) activity and fatty acid β-oxidation, which suppresses hepatic lipid catabolism. SCF^JFK^ is a real E3 ligase for ING5 and links the SCF^JFK^–ING5 axis to the development of metabolic syndrome obesity and non-alcoholic fatty liver disease (NAFLD) patients [55]; see Table 15.

Horvath et al. [56] noted that alteration of the secretion of insulin and insulin-like growth factor 1 (IGF1) led to decreased stem cell factor (SCF), the growth factor for cells of Cajal (ICC). These cells play a role as “pacemakers” in the stomach and act in response to gastric contractile activity. This may explain the depletion or disappearance of those cells in the muscular layers of the stomach in patients suffering from diabetic gastroparesis [56]; see Table 15.

Zhong et al. [57] reported that the peripheral plasma SCF level is higher in patients with non-dipper hypertension than those with dipper hypertension; note that subjects with a decline of less than 10% of nocturnal blood pressure (BP) compared to all day-time blood pressure values are defined as “non-dippers,” while those who suffer from a reduction above 10% of nocturnal BP compared to day-time levels are defined as “dippers”. Moreover, SCF was significantly correlated with 24-h mean systolic blood pressure (MSBP), 24-h mean diastolic blood pressure (MDBP), serum tumor necrosis factor-α (TNF-α), and interleukin 6 (IL-6) levels [57]; see Table 15.

Takematsu et al. [58] revealed, in their study, that the therapeutic potential effect of the transmembrane form of SCF (tmSCF) nanodiscs was a possible treatment option for peripheral ischemia. They treated rabbits with tmSCF nanodiscs, which were implemented into the ischemic limb of the rabbits. After eight weeks, they noticed well-expanded vascularity in the tmSCF nanodisc-treated group in comparison to the alginate-treated control. The results were determined through angiography. In addition to this, the histological analysis revealed a significantly elevated amount of large and small blood vessels in the ischemic muscles of the tmSCF nanodisc-treated group. Importantly, the researchers did not spot mast cell activation or inflammation in the rabbits [58]; see Table 15.

### 4.16. PDGF-BB in the Skin

Alexaki et al. [59] revealed, in their study, that platelet-derived growth factor-BB (PDGF-BB) influences the re-modeling and re-epithelialization of tissue [59]; see Table 16.

Das et al. [60] showed that wounds in diabetic patients who were cured with PDGF-BB and syndecan-4 proteoliposomes featured better-developed angiogenesis and re-epithelization versus treatment with PDGF-BB alone. Indeed, syndecan-4 proteoliposomes prompt the movement of keratinocytes originating from diabetic patients. In addition, syndecan-4 proteoliposomes sensitize keratinocytes to PDGF-BB activation. Apart from this, PDGF-BB with syndecan-4 proteoliposomes increased the M2 macrophage level and reduced the M1 macrophage amount, indicating that syndecan-4 may stimulate immunomodulation inside healing wounds [60]; see Table 16.

According to White et al. [61], triple therapy with HB-EGF-PlGF-2_123–144_, PDGF-BB-PlGF-2_123–144_, and VEGF-PlGF-2_123–144_ was effective against chronic non-healing diabetic wounds in a mouse model of type 1 diabetes [61]; see Table 16.

### 4.17. PDGF-BB in Skin Diseases

Pierce et al. [62] showed that treating chronic skin wounds with recombinant platelet-derived growth factor-BB (rPDGF-BB) prompted healing along with the formation of granulation tissue and fibroblast boosting. Moreover, in fibroblasts and capillaries, a raised level of PDGF-AA was noted. Both PDGF-AB and PDGF-BB were present in lower levels than PDGF-AA in healing wounds. Additionally, PDGF-beta receptors, known for their affinity to bind with PDGF-BB, are located in granulation tissue and in normal skin, highlighting their potential as a therapeutic approach for chronic wounds, thanks to exogenous rPDGF-BB [62]; see Table 17.

Jian et al. [63] demonstrated, in their research, that a novel bioactive peptide which showed better therapeutic effects and boosts wound healing was created after combination of the hydrogel process of the PDGF epitope VRKIEIVRKK with the peptide Nap-FFVLE. This new product may be engaged with a fibril-rich network and constitutes hydrogenation with adequate stability [63]; see Table 17.

Wu et al. [64] showed, in their results, that zeaxanthin (dihydroxy carotenoid) can be an effective inhibitor of the movement of fibroblasts activated by PDGF-BB and melanoma cells. Notably, PDGF not only acts as a chemotactic factor for dermal fibroblasts, but also takes part in the progression of melanoma. More precisely, zeaxanthin weakened PDGF-BB and mitogen-activated protein (MAP) kinase and melanoma-linked phosphorylation of PDGFR-beta in human skin fibroblasts [64]; see Table 17.

Sun et al. [65] proved that collagen fibers accompanied by PDGF-BB might be an effective booster for ulcer healing. Their study demonstrated that high bioactivity and high levels of PDGF-BB on the collagen membranes not only enhance collagen deposition and the formation of capillary walls but, most importantly, the re-epithelialization of dermal ulcer wounds as well [65]. Drela et al. [66] noted that the PDGF-BB level is elevated in the blood of patients suffering from diabetic foot syndrome (DFS). Consequently, a heightened PDGF-BB ratio can be linked with depleted limb ischemia, which is one of the pathomechanisms of DFS pathogenesis [66]. Conversely, according to Park et al. [67], PDGF-BB was found to be ineffective in stimulating wound healing in vivo in db/db mice [67]; see Table 17.

### 4.18. PDGF-BB in Psoriasis

Raynaud et al. [68] demonstrated that retinoic acid has the capacity to modify PDGF activity in psoriatic fibroblasts. This modulation is achieved through an impact on a post-receptor mechanism, rather than direct binding of the ligand to these cells. Precisely, retinoic acid influences psoriatic fibroblasts by increasing the mitogenic effect of PDGF in both lesional and non-lesional skin. Conversely, when applied to normal fibroblasts, retinoic acid treatment does not have any influence on the chemotactic and mitogenic activity of PDGF [68]; see Table 18.

### 4.19. PDGF-BB in Metabolic Syndrome

Tisato et al. [69] reported that metabolic syndrome is connected with a decrease in PDGF-BB, which is a confusing issue between fibrosis and the inflammation process in patients suffering from metabolic syndrome. In the same study, the authors did not find any correlation between PDGF-BB and any component linked to metabolic syndrome, such as blood pressure (systemic and diastolic) or disturbances in the concentration of glucose, triglycerides, insulin, creatinine, cholesterol, HDL, or low-density lipoprotein (LDL) [69]; see Table 19.

On the other hand, Shan et al. [70] documented an increase in the ratio of PDGF in sera of gestational diabetes mellitus (GDM) mice in comparison to non-gestational mice. Particularly, PDGF signaling directly led to β-cell abnormality during gestation. Interestingly, the serum PDGF level was negatively correlated with β-cell function in patients suffering from GDM. Moreover, it was noted that, after application of PDGF-BB, glucose tolerance was disturbed alongside the β-cell function, not even causing apoptosis in gestational mice in comparison to non-gestational mice. These results indicate that PDGF depletes the role of β-cells in gestation, perhaps through β-cell de-differentiation [70]; see Table 19.

Yeboah et al. [71] reported that a low plasma level of PDGF-BB is linked with prior cardiovascular issues in type 2 diabetes mellitus (T2DM). In particular, the mean plasma PDGF-BB concentration was elevated in a group without prior CVD events in comparison to the group with prior CVD disease. Moreover, their results may indicate that PDGF-BB plays an important role in macrovascular and microvascular complications in diabetes mellitus. Obviously, PDGF-BB can protect against those abnormalities by boosting the migration and proliferation of cells in vascular smooth muscle, which subsequently leads to a thicker fibrous cover, making the atheroma less prone to injury [71]; see Table 19.

Wang et al. [72] reported that PDGF-BB plays an essential role in the initiation and progression of diabetic nephropathy (DN). The urine level of PDGF-BB in DM can be a marker of early diagnosis of diabetic renal impairment. Moreover, urinary PDGF-BB was observed to be positively correlated with urine albumin excretion (UAE), triglyceride (TG), cholesterol, and low-density lipoprotein (LDL) and, at the same time, negatively correlated with creatinine clearance (Ccr) and HDL, and without significant correlation with glycohemoglobin A1c (HbA1c) [72]. Fagerudd et al. [73] revealed that patients suffering from insulin-dependent diabetes mellitus (IDDM) present an elevated urinary excretion of PDGF in comparison to healthy people. Based on this study, PDGF might be a predictor of the development of diabetic nephropathy [73]. Bessa et al. [74] also noted that PDGF-BB takes part in the initiation and progression of DN. Moreover, it is recognized as a prognostic factor for early impairment of renal function in DN. In addition, patients with diabetes dealing with macro- and micro-albuminuria had significantly elevated levels compared to those patients with normoalbuminuria. Moreover, urinary PDGF-BB was positively correlated with LDL, urinary albumin, and disease duration, but negatively correlated with creatinine clearance in patients suffering from diabetes mellitus. In summary, urinary PDGF-BB is strongly and independently linked with nephropathy in patients with diabetes [74]; see Table 19.

Kawano et al. [75] showed that the increase in aortic smooth muscle cells (SMCs) in diabetic rabbits and rats is higher than those in controls, which is linked with the over-expression of PDGF beta-receptors. Clearly, PDGF-BB boosts the growth of diabetic SMCs more than that of control SMCs. In addition, SMCs from diabetic rats exhibit higher expression of PDGF beta-receptor mRNA than SMCs from healthy rats. Moreover, in vivo, the aortic media of diabetic rabbits expressed PDGF beta-receptor mRNA, while that of non-diabetic rabbits did not. Thus, diabetic SMCs react to PDGF stimulation through over-expression of the PDGF beta-receptor gene [75]; see Table 19.

Rossi et al. [76] noted that the increase in plasma PDGF (PDGF-BB) in never-treated hypertension is an essential effect of platelet activation. Platelet extracts obtained from patients with hypertension featured boosted growth-promoting activity in vascular SMCs. Summarizing, the raised level of circulating PDGF might play an important role in the vascular re-build linked with hypertensive disease [76]; see Table 19.

Wang et al. [77] reported that high-fat diet feeding decreased the number of cardioprotective factors such as PDGF-BB, according to Vantler et al. [78], and eNOS in cardiac tissue. To the contrary, regular aerobic exercise activated PDGF-BB and eNOS signaling. In this study, the authors showed that regular aerobic exercise prompts cardioprotective effects by combating the obesity-associated inflammatory response [77]; see Table 19.

Rivera et al. [79] observed a reduction in PDGF-BB in young people suffering from obesity in comparison to healthy adolescents. Moreover, it was assumed by the authors that the alterations in PDGF-BB levels in adolescents were connected to obesity and were not related to the development of identifiable insulin resistance. In conclusion, PDGF-BB could be potentially used as a biomarker of IR in pubertal children dealing with obesity [79]; see Table 19.

### 4.20. M-CSF in Skin Diseases

Pellefigues et al. [80] stated that basophil-derived macrophage colony-stimulating factor (M-CSF) restricted the influx of pro-inflammatory molecules in the atopic dermatitis skin and, at the same time, boosted the expansion and function of pro-resolution M2-like macrophages, which are also responsible for efferocytosis [80]; see Table 20.

Li et al. [81], in their study, stated that stimulation of wound repairment and hair follicle regeneration was due to M-CSF activation of CD11b-positive myeloid cells in the murine epithelium [81]; see Table 20.

### 4.21. M-CSF in Psoriasis

Fuentelsaz-Romero et al. [82] showed that the expression of the M-CSF-dependent anti-inflammatory rate of CD209 pointed to only two macrophage groups (CD163^+^ CD209^low/−^ and CD163^+^ CD209^high^). CD209^+^ macrophages were more enriched in synovial tissue (ST) from healthy controls and patients dealing with psoriatic arthritis (PsA); however, both macrophage clusters were characterized by similar amounts of pro-inflammatory cytokines [82]; see Table 21.

Jadon et al. [83] noted that the serum concentration of M-CSF was significantly lower in PsA compared with both psoriatic patients without arthritis and healthy controls. According to the authors, M-CSF could be used as biomarker of arthritis in the psoriatic patient cohort [83]; see Table 21.

Cubillos et al. [84] demonstrated that peripheral blood mononuclear cells (PBMCs) stimulated by M-CSF in patients suffering from psoriatic arthritis affect the production of the higher proinflammatory cytokines levels. Moreover, the profile of secreted cytokine in response to vitamin D was different when compared to PBMCs from patients with only skin involvement and healthy controls [84]; see Table 21.

### 4.22. M-CSF in Metabolic Syndrome

Liu et al. [85] showed that M-CSF mRNA is up-regulated in the cells of the retina 2 weeks after the beginning of diabetes. Moreover, the vitreous amount of M-CSF is also increased in subjects suffering from proliferative diabetic retinopathy (PDR) in comparison to healthy ones. In summary, the signaling role of M-CSF may be a crucial factor in mediating neuron–glia communication in diabetic retinopathy [85]; see Table 22.

Ko et al. [86] revealed that a lack of M-CSF in mice caused milder vascular remodeling, endothelial dysfunction, and oxidative stress stimulated by deoxycorticosterone acetate (DOCA)-salt than +/+ and osteopetrosis (Op)/+ mice, indicating that inflammation might play a key role in DOCA-salt caused hypertension [86]; see Table 22.

Radaeva et al. [87] indicated that patients dealing with stage II essential hypertension and medical history lasting around 10–14 years were characterized by a higher concentration of M-CSF in their blood (*p* > 0.001). Interestingly, they may also have had an elevated changeability of the cardiac rhythm due to the M-CSF content in their blood. Moreover, M-CSF might serve as a marker of cardiovascular complications in the five-year risk period, such as myocardial infarction and acute cerebrovascular accident [87]; see Table 22.

Chung et al. [88] reported that forkhead box protein (FoxO1) is present in the M-CSF-derived (M2-like) macrophage sub-group and potentiates IL-10 expression on them. Additionally, their study highlighted that macrophages in conditions of hyperglycemia are transformed to the macrophage-inflammatory type through debilitation of the contribution of FoxO1 to the activation of IL-10 expression [88]; see Table 22.

Sugita et al. [89] reported, in their study, that there was no significant alteration in M-CSF amount in the fatty tissue of obese mice. Although a slight modification was observed—precisely, macrophage influx in the op/+ mice’s adipose tissue—it was pointed out that M-CSF does not play a significant role in macrophage enrollment in the adipose tissue of obese mice [89]; see Table 22.

Utsunomiya et al. [90] showed, in their study, that M-CSF treatment has no influence on serum total cholesterol in both hypercholesterolemic and diabetic rats. Additionally, implementing M-CSF minimized the increase of albumin outflow in urine in hypercholesterolemic diabetic rats with respect to diabetic rats with a normal level of cholesterol [90]; see Table 22.

Contrary to Utsunomiya et al. [90], Shimano et al. [91] reported that the total level of cholesterol was reduced in the plasma of Watanabe heritable hyperlipidemic (WHHL) rabbits treated with M-CSF. The authors concluded that the depletion of total plasma cholesterol level was caused by the decline of lipoproteins containing apo B 100, such as intermediate density lipoprotein (IDL), very low-density lipoprotein (VLDL), and LDL [91]; see Table 22.

Inoue et al. [92] showed retarded progression of atherosclerosis in response to M-CSF application. Moreover, the authors observed a decreased accumulation of cholesterol ester in the aortae of M-CSF-treated rabbits (0.60 +/− 0.32 mg/g tissue) in comparison to healthy rabbits (4.32 +/− 0.61 mg/g tissue). In summary, M-CSF prevented the progression of atherosclerosis in WHHL rabbits [92]. Donnelly et al. [93] also reported that treatment of rabbits with recombinant human M-CSF (rhM-CSF) caused the depletion of cholesterol in foam cells (macrophages containing cholesterol) [93]. On the other hand, Watanabe et al. [94] found that there was a great difference in the ratio of intimal to medial thickness (1.08 vs. 1.7, *p* < 0.01); precisely, the intimal thickness was 257 pm in the group treated with M-CSF and 328 pm in the healthy group. This means that M-CSF likely impacted the VSMC and, at the same time, alleviated atherosclerosis in those rabbits [94]; see Table 22.

### 4.23. VEGF in Skin

Leung et al. [95] proved that VEGF is a potent signaling molecule that plays a crucial role in angiogenesis—the process of forming new blood vessels. VEGF exerts its effects by binding to and activating specific tyrosine kinase receptors, VEGF receptors (VEGFRs), on endothelial cells. Upon VEGF stimulation, VEGFRs trigger a cascade of intracellular signaling events that promote endothelial cell proliferation, migration, differentiation, and survival, all of which are essential steps in angiogenesis [95]; see Table 23. 

Yano et al. [96] provided evidence that perifollicular angiogenesis is regulated by VEGF. Their findings suggest that increasing VEGF expression in the outer root sheath of hair follicles could be a promising approach for promoting hair growth [96]; see Table 23.

### 4.24. VEGF in Skin Diseases

The study by Choi et al. [97] provided evidence that VEGF is involved in the pathogenesis of systemic sclerosis (SSc), a chronic autoimmune disorder characterized by thickening and hardening of the skin with the involvement of internal organs. Their study found that high levels of VEGF were present in the blood of SSc patients, and that these levels were even higher in patients with diffuse SSc—a more severe form of the disease—than in limited SSc. Additionally, the severity of nailfold capillary loss, a common manifestation of SSc, was positively correlated with VEGF level. Nailfold capillary loss is a measure of the density of small blood vessels in the nailfold, which is associated with Raynaud’s phenomenon. They also found that the extent of skin sclerosis, as measured via the Rodnan skin score, was positively correlated with the VEGF level. The Rodnan skin score is a standardized method for assessing the severity of skin thickening in SSc patients. These findings suggest that VEGF may play a role in the development of SSc by promoting angiogenesis (i.e., the formation of new blood vessels). According to the authors, excessive angiogenesis is thought to contribute to the thickening and hardening of the skin in SSc [97]; see Table 24.

Tedeschi et al. [98] provided evidence that VEGF may play a role in the pathophysiology of chronic urticaria (CU). They found that VEGF plasma levels were significantly higher in CU patients compared to healthy controls. Additionally, VEGF levels were found to correlate with disease severity, with patients with more severe CU having higher VEGF levels. These findings suggest that VEGF may be involved in the development and progression of CU. They also found that eosinophils were the main cellular source of VEGF in CU lesional skin. Eosinophils are known to play a role in the inflammatory process that underlies CU [98]; see Table 24.

### 4.25. VEGF in Psoriasis

Young et al. [99] have provided evidence that VEGF is involved in the pathogenesis of psoriasis. They observed elevated levels of VEGF in psoriatic plaques, suggesting that VEGF may play a role in the abnormal growth of blood vessels, which is a hallmark of psoriasis. Additionally, VEGF polymorphisms may be associated with early-onset psoriasis. Retinoids, an established systemic therapy for psoriasis, have been shown to block VEGF production through the activator protein-1 (AP-1) site. They also found that PBMCs and epidermal keratinocytes (KCs) from patients with psoriasis demonstrate differential, genotype-dependent regulation of VEGF. Retinoic acid, a type of retinoid, was found to inhibit KCs’ production of VEGF in a genotype-dependent manner, while it stimulated the production of PBMCs regardless of VEGF genotype. These findings suggest that the regulation of VEGF by RA is complex and may be influenced by genetic factors [99]; see Table 25.

The case study by Akman et al. [100] suggested that bevacizumab—a monoclonal antibody that inhibits VEGF—may be a promising therapeutic agent for the treatment of psoriasis. Bevacizumab has been approved for the treatment of several types of cancer, including colon cancer. In the report by Akman et al. [100], a patient with metastatic colon cancer and psoriasis experienced complete remission of psoriasis during treatment with bevacizumab, suggesting that bevacizumab may have therapeutic benefits for psoriasis [100]; see Table 25.

### 4.26. VEGF in Metabolic Syndrome

Blann et al. [101] found that plasma VEGF levels were elevated in patients with hyperlipidemia, and that these levels could be reduced through lipid-lowering therapy. Additionally, patients with hyperlipidemia who received lipid-lowering therapy presented a significant reduction in plasma VEGF level. These findings suggest that VEGF may play a role in the pathogenesis of hyperlipidemia, and that lipid-lowering therapy may be an effective way to reduce VEGF levels. They also found that there was no significant correlation between plasma VEGF levels and the von Willebrand factor, a marker of endothelial damage. The increase in plasma VEGF levels in patients with hyperlipidemia does not appear to be related to endothelial damage [101]; see Table 26.

Facemire et al. [102] have provided evidence that VEGF plays a role in blood pressure regulation. The researchers found that administration of an antibody against the VEGF receptor VEGFR2 in normal mice resulted in a significant increase in blood pressure. This suggests that VEGF signaling is important for maintaining normal blood pressure. The researchers also found that VEGF increased the expression of nitric oxide synthase (NOS) and nitric oxide (NO) activity in the endothelium (i.e., the lining of blood vessels). NO is a signaling molecule that helps to relax blood vessels, which can lower blood pressure [102]; see Table 26.

### 4.27. TGF-α in the Skin

The study by Grellner et al. [103] provided evidence that transforming growth factor alpha (TGF-α) plays a critical role in wound healing processes, particularly by stimulating angiogenesis and the formation of new blood vessels. TGF-α also promotes cell restitution and proliferation, vasodilation, and the healing of both acute and chronic lesions [103]; see Table 27.

### 4.28. TGF-α in Skin Diseases

Koyama et al. [104] reported that TGF-α can act as an autocrine or paracrine growth factor on local sites of tumors involving epidermal growth factor (EGFR) receptors. For TGF-α to act in an endocrine fashion on its receptors in target cells a long distance from the stomach, such as those in the skin or mucosa of the esophagus, large quantities of TGF-α would have to be produced by tumor cells themselves to counteract the effect of dilution of the growth factors in circulation. Moreover, it was concluded by the researchers that high levels of serum TGF-α over a long time were a major cause of acanthosis nigricans as a cutaneous paraneoplastic syndrome [104]; see Table 28.

Partridge et al. [105] reported a high detection of TGF-α transcripts and EGFR in oral SCC, along with an inverse correlation between TGF-α protein and EGFR levels, suggesting that an autocrine growth factor loop plays a role in the growth of these tumors. This means that the tumor cells produce TGF-α, which binds to EGFR on the same cells, stimulating their growth. This autocrine growth loop is likely to be important for the development and progression of oral SCC. The higher levels of TGF-a in SCC compared to normal skin further support the role of TGF-α in oral SCC. This suggests that the production of TGF-α is increased in oral SCC cells, which may contribute to their uncontrolled growth [105]; see Table 28.

### 4.29. TGF-α in Psoriasis

Elder et al. [106] have provided evidence that TGF-α is over-expressed in psoriatic epidermis. The researchers found that TGF-α mRNA and protein levels were significantly higher in lesional psoriatic skin compared to non-lesional skin of psoriatic patients and to normal epidermis. These high levels of TGF-α in psoriatic epidermis are thought to contribute to the excessive proliferation of skin cells, which is a hallmark of psoriasis. TGF-α stimulates the growth of keratinocytes, the main cell type of the epidermis. This leads to the rapid turnover of skin cells, which results in the thick, scaly plaques of psoriasis [106]; see Table 29.

Higashiyama et al. [107] have provided evidence that the level of TGF-α is significantly higher in psoriatic-involved epidermis compared to the normal epidermis; in particular, the researchers found that the TGF-α level in psoriatic-involved epidermis was 4.62 times higher than that of the normal epidermis (*p* < 0.001). Therefore, the increased levels of TGF-α were involved in the induction or the maintenance of hyperproliferation of psoriatic epidermal keratinocytes [107]; see Table 29.

### 4.30. TGF-β in the Skin

Hirai et al. [108] reported, in their study, on the role of skin-derived signals in the maintenance of both epidermal-resident memory T cells (TRM cells) and circulating memory T cells (CMT cells) in the skin. The researchers found that antigen-specific CMT cells migrated into the skin following the resolution of skin vaccinia virus (VV) infection. However, in mice lacking αvβ6 and αvβ8 integrins—which are required for the activation of TGF-β by keratinocytes—there was a gradual loss of both E- or P-selectin-binding central and peripheral memory populations. This loss of CMT cells could be rescued by inhibiting skin entry, suggesting that skin-derived signals are important for the maintenance of CMT cells in the skin. The findings of this study suggest that skin-derived signals, including TGF-β, play a role in the maintenance of both TRM cells and CMT cells in the skin. This outcome could be used to develop new therapies for skin infections and other skin-related diseases [108]; see Table 30.

Schmid et al. [109] have provided evidence that transforming growth factor beta 3 (TGF-β3) plays a central role in epidermal maintenance, and that induction of transforming growth factor-beta 1 (TGF-β1) in migrating keratinocytes is a key event required for the successful re-epithelialization of skin wounds. The researchers found that TGF-β3 was more potent than TGF-β1 and transforming growth factor beta 2 (TGF-β2) in inhibiting the growth of primary human keratinocytes. Additionally, the induction of TGF-β1 in migrating keratinocytes was associated with an increase in keratinocyte proliferation and migration. These findings suggest that TGF-β3 and TGF-β1 play distinct roles in wound healing [109]; see Table 30.

### 4.31. TGF-β in Skin Diseases

Santiago et al. [110] have provided evidence that topical application of P144—a peptide inhibitor of TGF-β1—is an effective and safe treatment for skin fibrosis. The researchers found that topical application of P144 significantly reduced skin fibrosis and soluble collagen content in a well-characterized model of human scleroderma. Importantly, P144 was effective in both preventing and treating skin fibrosis [110]; see Table 31.

Denton et al. [111] were the first to evaluate a systemically administered and repeatedly dosed anti-TGFβ1 drug, CAT-192, in patients with early diffuse cutaneous SSc. They found that CAT-192, in doses up to 10 mg/kg, showed no evidence of efficacy in terms of improving clinical or biochemical outcomes [111]; see Table 31.

### 4.32. TGF-β in Psoriasis

Wataya-Kaneda et al. [112] have provided evidence that TGF-β2 may play a role in the pathogenesis of psoriasis. The researchers found that TGF-β2 was decreased in the psoriatic epidermis compared to the normal epidermis. As TGF-β2 is a strong growth inhibitor for human keratinocytes, this result suggests that a decrease in TGF-β2 may be involved in the excessive proliferation of keratinocytes, which is a hallmark of psoriasis. In addition to its role in keratinocyte proliferation, TGF-β2 is also a potent immunosuppressive agent. This suggests that the decrease in TGF-β2 in psoriatic epidermis may also contribute to the inflammation that is characteristic of the disease [112]; see Table 32.

Flisiak et al. [113] proved that TGF-β1 may be a biomarker for psoriasis activity. The researchers found that plasma TGF-β1 levels were significantly correlated with the PASI, a measure of disease severity. Additionally, TGF-β1 levels in skin scales were significantly correlated with sedimentation rate (a measure of inflammation) and disease duration. These findings suggest that TGF-β1 may play a role in the pathogenesis of psoriasis, and that it may be a useful biomarker for monitoring disease activity [113]; see Table 32.

### 4.33. TGF-β in Metabolic Syndrome

Lin et al. [114] reported that the mothers against decapentaplegic homolog 2 (SMAD2) and transforming growth factor beta receptor 2 (TGFBR2) genes may be associated with MS and its components. The researchers found that single-nucleotide polymorphisms (SNPs) in the SMAD2 and TGFBR2 genes were associated with an increased risk of MS, both independently and through complex gene–gene interactions. Additionally, the researchers found that TGF-β signaling pathway-associated genes were associated with the components of MS [114]; see Table 33.

Herder et al. [115] have given evidence that elevated serum concentrations of TGF-β1 indicate an increased risk for T2DM. The researchers found that serum levels of TGF-β1 were significantly higher in individuals with T2DM compared to individuals without T2DM. Additionally, the researchers found that an increase in serum TGF-β1 level preceded the development of T2DM by several years [115]; see Table 33.

### 4.34. EGF in the Skin

Cohen et al. [116] showed that epidermal growth factor (EGF) is a small protein that binds to a specific receptor, called the epidermal growth factor receptor (EGFR), on the surface of cells. When EGF binds to EGFR, it activates a signaling pathway that leads to a variety of cellular responses, including cell growth, proliferation, differentiation, and survival [116]; see Table 34.

Bhora et al. [117] showed that EGF resulted in the most epithelial outgrowth, followed by fibroblast growth factor (FGF) and insulin-like growth factor-1 (IGF-1). This result suggests that EGF plays a leading role in promoting cell proliferation and migration, which are essential processes in wound healing. EGF is a particularly potent stimulator of epithelialization, which is the process of new skin cells forming over a wound. This is likely due to its ability to activate signaling pathways that promote cell proliferation and differentiation [117]; see Table 34.

### 4.35. EGF in Skin Diseases

Choi et al. [118] investigated the effects of EGF on the expression of inflammatory cytokines and antimicrobial peptides (AMPs) in human epidermal keratinocytes (HEKs) treated with heat-inactivated *S. aureus* (HKSA) in vitro and 2,4-dinitrochlorobenzene-induced AD-like skin lesions in Nc/Nga mice. Furthermore, EGF increased the expression of human β defensin-2 (an AMP) in HEKs and murine β defensin-3 in mice. In mice, both EGF and pimecrolimus—a topical calcineurin inhibitor—groups showed reduced erythema, inflammation, and expression of thymic stromal lymphopoietin (TSLP), a cytokine that plays a role in the pathogenesis of AD. These findings suggest that EGF could be a potential topical treatment for AD. The anti-inflammatory and antimicrobial properties of EGF make it a promising candidate for the treatment of this chronic skin condition [118]; see Table 35.

Paik et al. [119] investigated the potential of a topical EGF-liposomal solution to protect hair follicles from cyclophosphamide-induced alopecia (CIA) in C57BL/6 mice. They found that topical EGF application induced a catagen-like stage in hair follicles, which made them more resistant to chemotherapy-mediated damage. Additionally, EGF treatment favored primary hair recovery through the dystrophic anagen pathway after CIA [119]; see Table 35.

### 4.36. EGF in Psoriasis

Flisiak et al. [120] observed that the mean serum EGF concentration in patients with psoriasis was higher than in controls; however, the difference was not significant. The mean serum concentration of soluble epidermal growth factor receptor (sEGFR) was significantly lower than in controls. Serum levels of EGF showed a significant positive correlation, and EGFR showed a significant negative correlation with PASI. No correlation was observed between PASI and EGF content in scales or between EGF and sEGFR levels. Serum EGF concentrations reached the highest mean level in patients with PASI > 20, and this was significantly higher than the mean in the group with PASI < 10. Mean sEGFR serum concentrations remained significantly lower than those of controls, regardless of disease severity. The positive correlation between EGF level and PASI suggests that EGF may be a marker of disease severity [120]; see Table 36. 

Nanney et al. [121] revealed an enhanced binding capacity for EGF in the upper layers of the epidermis in psoriatic skin compared to normal thin or thick skin. It was observed that, when measured on a protein basis, [125I] EGF binding was significantly increased in psoriatic epidermis compared to normal epidermis of similar thickness. The retention of EGF receptors in the non-mitotic stratum spinosum and parakeratotic stratum corneum may reflect the abnormal and incomplete differentiation that occurs in active psoriatic lesions [121]; see Table 36.

### 4.37. EGF in Metabolic Syndrome

Kyohara et al. [122] found that sEGFR levels were correlated with various factors related to hepatic insulin resistance, including fasting blood glucose level, HOMA-IR, HbA1c level, HDL-cholesterol level, and FIB-4 index. These findings suggest that soluble EGFR may play a role in the development of hepatic insulin resistance, which is a major risk factor for T2DM and other metabolic diseases. Soluble EGFR is a circulating form of EGFR, a protein that plays a role in cell growth and proliferation. The authors suggested that soluble EGFR may be involved in the development of hepatic insulin resistance by promoting the growth and proliferation of liver cells [122]; see Table 37.

Belmadani et al. [123] showed that elevated EGFR phosphorylation may play a role in the dysfunction of coronary arteries and mesenteric resistance arteries (MRAs) in diabetic db/db mice. This dysfunction is characterized by increased myogenic tone, impaired endothelium-dependent relaxation, and structural re-modeling. Treatment with an EGFR inhibitor, AG1478, normalized these abnormalities, suggesting that EGFR is a potential target for overcoming diabetic small artery complications [123]; see Table 37.

### 4.38. IGF in the Skin

Tavakkol et al. [124] have provided valuable insights into the expression and function of insulin-like growth factor 1 (IGF-1) and its receptor in human skin. IGF-1 is produced by fibroblasts, melanocytes, and keratocytes, but not by keratinocytes themselves. This implies that keratinocytes may rely on paracrine signaling from other cell types for IGF-1 stimulation. Keratinocytes are responsive to IGF-1 due to the abundance of IGF-1 receptor mRNA. This suggests that keratinocytes are a primary target of IGF-1 signaling in the skin, and IGF-1 directly stimulates keratinocyte proliferation. GH, on the other hand, may indirectly affect keratinocyte proliferation by inducing IGF-1 production in other cells. These findings highlight the importance of IGF-1 signaling in regulating keratinocyte proliferation and maintaining skin homeostasis. Further studies are warranted to elucidate the precise mechanisms by which IGF-1 controls keratinocyte growth and differentiation [124]; see Table 38.

Lewis et al. [125] showed that IGF-1 produced by human fibroblasts is essential for the appropriate ultraviolet B (UVB) response of keratinocytes. This means that, when fibroblasts are unable to produce IGF-1, keratinocytes are not able to properly respond to UVB radiation. The expression of IGF-1 is silenced in senescent fibroblasts in vitro. This suggests that the age-related decline in IGF-1 production may be due to senescence of dermal fibroblasts. IGF-1 expression is also silenced in geriatric dermis in vivo. This confirms that the age-related decline in IGF-1 production is not limited to cultured cells, but also occurs in human skin. The diminished IGF-1 expression in geriatric skin correlates with an inappropriate UVB response in geriatric volunteers [125]; see Table 38.

### 4.39. IGF in Skin Diseases

Rahaman et al. [126] studied the role of IGF-1 in acne. The mean plasma IGF-1 level was significantly higher in acne cases than in non-acne controls, suggesting that IGF-1 may play a role in the development of acne. Furthermore, the plasma IGF-1 level was positively correlated with the severity of acne, suggesting that higher IGF-1 levels may be associated with more severe acne [126]; see Table 39.

Tan et al. [127], in their study, found that IGF-1 expression was significantly higher in the affected mucosa of OLP patients than in healthy mucosa. This increased expression of IGF-1 correlated with an increased concentration of T cells in the tissue, suggesting that IGF-1 may stimulate the proliferation of T cells, which may contribute to the pathogenesis of OLP [127]; see Table 39.

### 4.40. IGF in Psoriasis

Miura et al. [128] found that IGF-1 mRNA expression was significantly higher in psoriatic fibroblasts than in control fibroblasts (CF). However, no significant difference in IGF-I mRNA was observed between non-lesional psoriatic fibroblasts and CF. These results suggest that dermal fibroblasts may contribute to the epidermal hyperplasia of psoriasis by promoting keratinocyte proliferation through IGF-1, whose secretion could be modulated by inflammatory cytokines [128]; see Table 40.

In their study, El-Komy et al. [129] provided valuable insights into the role of IGF-1 in psoriasis and the potential therapeutic effects of methotrexate and PUVA therapy. IGF-1 and its mRNA levels were significantly elevated in lesional skin of psoriatic patients compared to controls. Both methotrexate and PUVA treatment were associated with a significant decrease in both PASI scores and lesional IGF-1 after 10 months of treatment. This suggests that both treatments may be effective in reducing psoriasis severity and IGF-1 levels. The down-regulation of IGF-1 following methotrexate or PUVA treatment may be due to a decrease in pro-inflammatory cytokines, inflammatory cellular infiltration, or an effect on local fibroblast activity and proliferation [129]; see Table 40.

### 4.41. IGF in Metabolic Syndrome

The study by Saydah et al. [130] investigated the association between MS components and circulating levels of IGF-1, IGF-binding protein 3 (IGF-BP3), and the IGF-1/IGF-BP3 ratio. Their findings revealed that each component of MS was associated with lower levels of IGF-1, IGF-BP3, and the IGF-I/IGF-BP3 ratio. Additionally, higher insulin levels were observed in participants with each metabolic syndrome component [130]; see Table 41.

Efrastadias et al. [131] conducted a study that investigated the relationship between IGF-1, C-reactive protein (CRP), MS, and CVD. Their findings revealed that both low IGF-1 and high CRP levels were associated with an increased number of MS components. Additionally, a negative correlation was observed between CRP and IGF-1 levels, suggesting that an increase in CRP may contribute to a reduction in the IGF-1 concentration [131]; see Table 41.

### 4.42. The Role of Growth Factors in Response to Biological Treatment of Psoriasis

#### 4.42.1. TNF Inhibitors and Their Influence on BMI

Tan et al. [132] revealed that tumor necrosis factor inhibitors (TNFI) were associated with weight gain in some patients [132]. Hojgaard et al. [133] reported that the exact mechanism of TNFI-induced weight gain is not fully understood, but it is thought to be related to changes in appetite and metabolism. The metabolic implications of TNF-α cytokine, as a regulatory factor, may contribute to the weight gain associated with TNFI treatment for psoriasis. The accumulation of excess fat—particularly visceral fat—can increase the risk of cardiovascular complications, such as heart disease and stroke. This is particularly concerning in the context of psoriasis, as psoriasis itself is associated with an increased risk of CVD. The additional weight gain associated with TNFI therapy can further exacerbate this risk [133]. Naldi et al. [134] reported a statistically significant association between BMI and the likelihood of achieving PASI-75 in patients with psoriasis treated with TNFI therapy. Patients with obesity (BMI ≥ 30) were less likely to achieve PASI-75 than patients with normal weight (BMI = 20–24). Specifically, the adjusted odds ratio for achieving PASI-75 in obese patients was 0.73 (95% CI = 0.58–0.93) at 8 weeks and 0.62 (95% CI = 0.49–0.79) at 16 weeks [134]; see Table 42.

#### 4.42.2. IL-17A Inhibitors and Their Influence on BMI

Takamura et al. [135] have provided valuable insights into the potential weight-neutral effects of secukinumab compared to other TNFI therapies (particularly infliximab). Their findings suggest that secukinumab may offer a more favorable option for patients with psoriasis who are also concerned about weight management [135]; see Table 43.

Egeberg et al. [136] found that ixekizumab was associated with no significant changes in body weight or BMI after 12 weeks of treatment. This is in contrast to other TNFI therapies, such as infliximab, which have been shown to cause weight gain [136]; see Table 43.

Reich et al. [137] studied the effect of body weight on the response to ixekizumab, and showed that both efficacy and safety profiles were similar regardless of the body weight of patients. In addition, no significant differences in PASI 75 response rates were observed across body weight categories [137]; see Table 43.

Similarly, in the study by Reich et al. [138], it was found that the efficacy and safety of ixekizumab were similar in patients of all body weights. This is an important finding, as it suggests that ixekizumab may be an effective and safe treatment option for patients with psoriasis, regardless of their body weight. At week 12, 75% of patients in all body weight categories who received ixekizumab achieved a PASI 75 response, which indicates a 75% improvement in psoriasis symptoms at least. The safety profile of ixekizumab was also similar for all body weight categories. These findings suggest that ixekizumab is an effective and safe treatment option for patients with psoriasis, regardless of their body weight [138]; see Table 43.

In the study by Piros et al. [139], it was found that treatment with IL-17A inhibitors in severe psoriasis did not significantly change body composition parameters such as BMI. The median baseline BMI remained constant (at 32.8 kg/m²) after six months of treatment. This finding is consistent with the above-mentioned studies that investigated the effects of IL-17A inhibitors on body weight and body composition [139]; see Table 43.

#### 4.42.3. IL-12/23 Inhibitors and Their Influence on BMI

The study by Gisondi et al. [139] highlighted the potential advantages of ustekinumab over both TNFI and IL-17A inhibitors in terms of body weight and metabolism. The authors suggested that ustekinumab may offer a more balanced approach, with a neutral effect on body weight and metabolism, improved safety and tolerance compared to TNFI, and efficacy that is less affected by BMI compared to IL-17A inhibitors [139]; see Table 44. 

Conversely, the long-term extensions of the PHOENIX clinical trials, as reported by Lebwohl et al. [140] and Papp et al. [141], demonstrated a decrease in the efficacy of ustekinumab in patients with higher BMI values. Specifically, the studies found that patients with a BMI greater than 25 kg/m^2^ had a lower response rate to ustekinumab compared to patients with a lower BMI. This finding suggests that obesity may be a factor that influences the effectiveness of ustekinumab in treating psoriasis [140,141]; see Table 44.

The findings of Young et al. [142] and the ACCEPT trial highlight the potential impact of weight on the efficacy of ustekinumab in treating psoriasis. These studies suggest that patients with a higher body weight may benefit from a weight-adjusted dosing strategy in order to achieve optimal treatment outcomes. In the study by Young et al., doubling the ustekinumab dose from 45 mg to 90 mg resulted in a significant 20% increase in PASI90 response rates among patients weighing over 100 kg. This demonstrated that higher doses of ustekinumab may be more effective in treating psoriasis in heavier individuals. Similarly, the ACCEPT trial indicated that patients weighing over 100 kg had significantly lower PASI scores when treated with ustekinumab compared to those treated with etanercept. This further supports the notion that ustekinumab may be more effective in patients with high BMI [142]; see Table 44.

Zweegers et al. [143] concluded that a higher BMI is a predictor for discontinuation due to ineffectiveness in etanercept and ustekinumab modalities. Moreover, female sex was a consistent predictor for discontinuation due to side-effects in all three outpatient biologics (i.e., adalimumab, etanercept, ustekinumab) [143]; see Table 44.

## 5. Conclusions

Psoriasis is frequently accompanied by metabolic syndrome or its components. In the present study, we presented the current state of knowledge on the potential mechanisms that may be involved in the development of both psoriasis and components of metabolic syndrome.

The mechanisms by which these diseases are connected are not fully known, despite numerous studies having been carried out to date. In both diseases, the role of growth factors is complex and multi-faceted, impacting various aspects of inflammation, tissue re-modeling, cell proliferation, and metabolic regulation. Common pathways also include angiogenesis, which is observed in both psoriatic skin lesions and adipose tissue in metabolic syndrome. Dysregulation of the immune system is the common thread in both conditions, and growth factors can influence immune cell activation and function. While these pathways overlap, it is important to note that the specific mechanisms and the relative contributions of growth factors vary between the two conditions.

The fact that psoriatic patients dealing with obesity were characterized by worse response to biological agents, including tumor necrosis factor inhibitors, is also a significant result which should be further examined.

Further research is needed to fully elucidate their detailed mechanisms, possible clinical implications, benefits, and potential therapeutic implications under these conditions.

## Figures and Tables

**Table 1:** Summary of the studies on the role of b-FGF in the skin.

Author	Year	Population	Key Observation
The Role of b-FGF in the Skin			
Takehara [12]	2000	Mice	b-FGF maintains skin fibrosis.
Makino et al. [13]	2010	Cultured HDFs	b-FGF increased the number of HDFs.

HDFs—human dermal fibroblasts, b-FGF—basic fibroblast growth factor, JNK—Jun N-terminal kinase, MEK—mitogen-activated protein kinase, ERK1/2—extracellular signal-regulated kinase 1/2, JNK1—Jun N-terminal kinase 1, siRNA—small interfering ribonucleic acid, ERK1—extracellular signal-regulated kinase 1, ERK2—extracellular signal-regulated kinase 2.

**Table 2:** Summary of the studies on the role of b-FGF in skin diseases.

Author	Year	Population	Key Observation
The Role of b-FGF in Skin Diseases			
Song et al. [14]	2016	Mouse NIH3T3 fibroblast cell line	b-FGF promotes cell migration, which is an important factor in the wound healing process.
Nakamizo et al. [15]	2012	C57BL/6 (B6) and BKS.Cg– + Lepr db/+ Lepr db/Jcl mice (DB mice), NHEK	b-FGF enhances keratinocyte proliferation, which repairs the skin barrier disruption.
Qu et al. [16]	2018	Bovine tendons	b-FGF showed better-organized epidermal regeneration in the wound’s vascular networks.
Wu et al. [17]	1994	Young adult female New Zealand white rabbits (New Franken Inc., New Fanken, WI, USA)	b-FGF is ineffective in the setting of ischemia.
Richard et al. [18]	1995	17 diabetic patients with diabetes and dealing with chronic neuropathic ulcers	Topical b-FGF yielded no improvement in the healing of diabetic ulcers.

NHEK—normal human epidermal keratinocytes, KGF—keratinocyte growth factor.

**Table 3:** Summary of the studies on the role of b-FGF in psoriasis.

Author	Year	Population	Key Observation
The Role of b-FGF in Psoriasis			
Watanabe et al. [19]	2023	10 healthy controls, 18 patients with PsV, 24 patients with PsA, 13 patients with GPP	The serum b-FGF level is positively correlated PASI in GPP patients.
Sharpe et al. [20]	1989	Human keratinocytes and endothelial cells	Cyclosporine A inhibits proliferation of endothelial cells and keratinocyte proliferation mediated by b-FGF.
Przepiera-Bedzak et al. [21]	2013	98 patients, 80 with PsA, 18 with SAPHO syndrome	No significant correlation between clinical presentation and b-FGF.

PsV—psoriasis vulgaris, PsA—psoriatic arthritis, GPP—generalized pustular psoriasis, SAPHO—synovitis, acne, pustulosis, hyperostosis, osteitis; FGF-a—acidic fibroblast growth factor, PASI—Psoriasis Area and Severity Index.

**Table 4:** Summary of the studies on the role of b-FGF in metabolic syndrome.

Author	Year	Population	Key Observation
The Role of b-FGF in Metabolic Syndrome			
Ivannikova [22]	2015	134 patients with CHD and T2DM, including 38 patients with ACS	A significant two-fold increase in b-FGF in patients dealing with CHD, DM, and ACS.

CHD—coronary heart disease, DM—type 2 diabetes mellitus, ACS—acute coronary syndrome.

**Table 5:** Summary of the studies on the role of HGF in the skin.

Author	Year	Population	Key Observation
The Role of HGF in the Skin			
Qin et al. [23]	2017	Skin from 12 healthy people	HGF, mainly present in human skin dermal fibroblasts, is in charge of collagen production.
Gron et al. [24]	2002	Periodontal ligament	The raised level of HGF production can influence the proliferation and migration of junctional epithelium and the development of periodontal disease.
Recio et al. [25]	2002	Transgenic mouse line MH37	Enhanced activity of HGF/c-MET- may elevate their invasive capacity, boost the proliferation of melanoma cells, and prevent melanoma cells from apoptosis.
Zeng et al. [26]	2002	Human HNSCC cell lines	Elevated amounts of HGF may inhibit apoptosis of head and neck SCC.

ROL—retinoid, TGF-β—transforming growth factor β1, KGF—keratinocyte growth factor, c-MET—mesenchymal–epithelial transition factor, SCC—squamous cell carcinoma, HNSCC—Head and neck squamous cell carcinoma.

**Table 6:** Summary of the studies on the role of HGF in skin diseases.

Author	Year	Population	Key Observation
The Role of HGF in Skin Diseases			
Nicu et al. [27]	2021	Tissue samples from hair transplant surgeries	dWAT controls pigmentation and human hair growth through HGF secretion.
Bevan et al. [28]	2004	C57BL/KsOlaHsd db/db mice	HGF/SF affects and sustains and affects all essential cellular processes that are in charge of wound repair.
Otsuka et al. [29]	1998	Transgenic mice	HGF is also expressed in melanoma cells.

dWAT—dermal white adipose tissue.

**Table 7:** Summary of the studies on the role of HGF in psoriasis.

Author	Year	Population	Key Observation
The Role of HGF in Psoriasis			
Meng et al. [30]	2021	Mice	HGF in psoriatic mice lesions alleviates the erythema, scaling, and thickening.
Takahashi et al. [31]	2009	Keratinocytes from normal skin	Activation of TNF-alpha raised HGF, but the growth of HGF did not differ much between psoriatic skin and normal skin.

DPSCs—dental pulp stem cells, CK17—cytokeratin 17, IFN-γ—interferon-gamma, RORγt—retinoic acid-related orphan receptor-γt.

**Table 8:** Summary of the studies on the role of HGF in metabolic syndrome.

Author	Year	Population	Key Observation
The Role of HGF in Metabolic Syndrome			
Balaban et al. [32]	2006	26 patients with NASH, 13 controls	Serum HGF levels are strongly associated with IR and all components of MS.
Hiratsuka et al. [33]	2005	1474 healthy people	HGF levels are significantly related to HDL, waist circumference, and liver enzymes.
Faber et al. [34]	2013	A cohort of 1251 patients with manifest vascular disease	VAT tissue is mainly associated with level of HGF.
Sakaue et al. [35]	2023	2223 random patients	Improvement in PA levels is linked with depleted HGF levels and CVD development.
Tsukagawa et al. [36]	2013	1492 people	HGF level is also significantly associated with the progress of IR.
Sanchez-Escinales et al. [37]	2015	Double-transgenic mice	Muscle expression of HGF prevents obesity-mediated muscle IR and enhances glucose tolerance.
Motone et al. [38]	2004	654 people	C/A polymorphism in intron 13 of the HGF gene is connected with susceptibility to essential hypertension in lean or female subjects.

NASH—non-alcoholic fatty liver disease, IR—insulin resistance, VAT—Visceral adipose tissue, PA—physical activity, CVD—cardiovascular disease, BMI—body mass index.

**Table 9:** Summary of the studies on the role of NGF-β in skin diseases.

Author	Year	Population	Key Observation
The Role of NGF-β in Skin Diseases			
Sun et al. [39]	2010	Rabbit dermal ischemic ulcer model	NGF-β boosts ulcer healing.
Sari et al. [40]	2021	564 residents of long term care facility in Indonesia	NGF-β is associated with presence of ich in older adults.
Solinski et al. [41]	2021	11 people(6 males, 5 females)	Patients suffering from chronic itch are characterized with raised levels of NGF-β.
Peng et al. [42]	2013	75 patients with mastocytosis, 50 healthy patients	NGF-β is correlated with tryptase levels.

NGF-β—nerve growth factor-β, MCs—mast cells.

**Table 10:** Summary of the studies on the role of NGF-β in psoriasis.

Author	Year	Population	Key Observation
The Role of NGF-β in Psoriasis			
Baerveldt et al. [43]	2021	11 patients (5 male, 6 female)	NGF-β, after 4 weeks of ustekinumab injection, is significantly depleted.
Raychaudhuri et al. [44]		12 psoriatic patients, 4 healthy patients	Keratinocytes in psoriatic patients produce high levels of NGF-β.

**Table 11:** Summary of the studies on the role of NGF-β in metabolic syndrome.

Author	Year	Population	Key Observation
The Role of NGF-β in Metabolic Syndrome			
Molnar et al. [45]	2020	Postmenopausal women (n = 66), Obese women (n = 32), Control women (n = 35)	Obese and post-menopausal women have higher NGF-β levels.
Sisman et al. [46]	2014	Adult male Wistar albino rats	Lower NGF-β level is connected with an increase of apoptosis and testicular damage in diabetic rats.
Ueyama et al. [47]	1995	Okamoto–Aoki strain of rats	Lowered manufacture of NGF-β from VSMCs may be responsible for the hypotrophy of sympathetic nerve cells in GH rats.
Selavaraju et al. [48]	2013	40 normal weight, 20 overweight, 16 obese children aged 6–10	NGF-β is significantly raised in obese children.

VSMCs—vascular smooth muscle cells, GH—genetically hypertensive.

**Table 12:** Summary of the studies on the role of SCF in the skin.

Author	Year	Population	Key Observation
The Role of SCF in the Skin			
Franke et al. [49]	2023	Human foreskin tissue	TF CREB acts as a crucial intermediary in SCF-triggered KIT activation of human skMCs.

CREB—cAMP response element binding protein, SCF—stem cell factor, skMCs—skin mast cells, TF—transcription factor, KIT—KIT Proto-Oncogene, Receptor Tyrosine Kinase.

**Table 13:** Summary of the studies on the role of SCF in skin diseases.

Author	Year	Population	Key Observation
The Role of SCF in the Skin Diseases			
Yamanaka-Takaichi et al. [50]	2023	9 SK patients	SCF expression in SK is higher in comparison to the marginal lesion.

SK—Seborrheic keratosis.

**Table 14:** Summary of the studies on the role of SCF in psoriasis.

Author	Year	Population	Key Observation
The Role of SCF in Psoriasis			
Cho et al. [51]	2017	Female C57BL/6 mice	High expression of SCF in mice keratinocytes and HaCaT cells promotes the accumulation of epidermal mast cells.
Yamamoto et al. [52]	2000	20 patients with psoriasis vulgaris	SCF level is elevated in patients with psoriasis vulgaris in comparison to healthy people.

HaCaT—cultured human keratinocyte, SCF—stem cell factor.

**Table 15:** Summary of the studies on the role of SCF in metabolic syndrome.

Author	Year	Population	Key Observation
The Role of SCF in Metabolic Syndrome			
Wang et al. [53]	2020	C57BL/6J wild-type control mice, K14cre transgenic mice Scf^fl/fl^ mice	Skin wound repairment is delayed in metabolic syndrome and is linked with SCF depletion in keratinocytes.
Jialal et al. [54]	2010	Patients with MS (n = 36), controls (n = 38)	Obese males have a decreased level of SCF in plasma.
He et al. [55]	2021	JFK transgenic mice	The SCF^JFK^–ING5 axis suppresses hepatic lipid catabolism.
Horvath et al. [56]	2006	Young adult and 9–18-day-old BALB/c mice	ICC depletion in diabetes was accompanied by smooth-muscle atrophy and reduced SCF.
Zhong et al. [57]	2017	116 non-dipper patients, 131 dipper patients	SCF correlates significantly with MSBP, MDBP, TNF-α, and IL-6 levels.
Takematsu et al. [58]	2023	Rabbits with hyperlipidemia and diabetes	Significantly raised level of large and small blood vessels in the ischemic muscles of the tmSCF nanodisc-treated group of rabbits with hyperlipidemia and diabetes.

VEGF—vascular endothelial growth factor, ICC—cells of Cajal, MSBP—mean systolic blood pressure, MDBP—mean diastolic blood pressure, TNF-α—tumor necrosis factor-alpha, IL-6—interleukin 6, tmSCF—transmembrane form of SCF.

**Table 16:** Summary of the studies on the role of PDGF-BB in the skin.

Author	Year	Population	Key Observation
The Role of PDGF-BB in the Skin			
Alexaki et al. [59]	2012	7 healthy people	PDGF-BB influences the re-modeling and re-epithelialization of tissue.
Das et al. [60]	2016	Human keratinocytes from diabetic and healthy donors	Diabetic patients who are treated with PDGF-BB and syndecan-4 proteoliposomes have better angiogenesis and re-epithelization rate versus those who receive treatment with PDGF-BB alone.
White et al. [61]	2021	Non-obese diabetic mice, mice without diabetes	HB-EGF-PlGF-2_123–144_, PDGF-BB-PlGF-2_123–144_, and VEGF-PlGF-2_123–144_ can be an effective therapy for chronic non-healing diabetic wounds.

PDGF-BB—platelet-derived growth factor-BB, VEGF-PlGF-2_123–144_—vascular endothelial growth factor-A, PDGF-BB-PlGF-2_123–144_—platelet-derived growth factor-BB, HB-EGF-PlGF-2_123–144_—heparin-binding epidermal growth factor.

**Table 17:** Summary of the studies on the role of PDGF-BB in skin diseases.

Author	Year	Population	Key Observation
The Role of PDGF-BB in Skin Diseases			
Pierce et al. [62]	1995	15 rPDGF-BB-treated, 13 placebo-treated patients	rPDGF-BB prompts healing of chronic skin wounds.
Jian et al. [63]	2022	C57BL/6 mice	PDGF epitope VRKIEIVRKK with the peptide Nap-FFVLE boosts the process of wound healing.
Wu et al. [64]	2010	Human foreskin fibroblast cell line, human metastatic melanoma cell line	PDGF, as a chemotactic factor for dermal fibroblasts, may also be responsible for the progression of melanoma.
Sun et al. [65]	2007	Young adult New Zealand white rabbits	PDGF-BB can be an effective booster for ulcer healing.
Drela et al. [66]	2014	75 diabetic patients with DFS	PDGF-BB heightened level is linked with reduced limb ischemia.
Park et al. [67]	2014	db/db mice (type 2 diabetic mouse model)	PDGF-BB does not manage to stimulate the process of wound healing.

rPDGF-BB—recombinant platelet-derived growth factor-BB, DFS—diabetic foot syndrome; MAP—mitogen-activated protein kinase.

**Table 18:** Summary of the studies on the role of PDGF-BB in psoriasis.

Author	Year	Population	Key Observation
The Role of PDGF-BB in Psoriasis			
Raynaud et al. [68]	1991	6 healthy people, 6 people with psoriasis	Retinoic acid can inflect the PDGF activity in psoriatic fibroblasts.

**Table 19:** Summary of the studies on the role of PDGF-BB in metabolic syndrome.

Author	Year	Population	Key Observation
The Role of PDGF-BB in Metabolic Syndrome			
Tisato et al. [69]	2013	40 obese patients	Metabolic syndrome is connected with the decrease in PDGF.
Shan et al. [70]	2019	77 patients with GDM 69 healthy and pregnant without GDM	PDGF signaling leads directly to pancreatic β-cell abnormality during gestation.
Yeboah et al. [71]	2007	80 patients with T2DM	Low plasma level of PDGF-BB is linked with prior cardiovascular issues in T2DM.
Wang et al. [72]	2009	65 patients with T2DM	PDGF-BB plays an essential role in the initiation and progression of diabetic nephropathy (DN).
Fagerudd et al. [73]	1997	104 patients with IDDM, 30 controls	Patients suffering from IDDM have an elevated amount of urinary excretion of PDGF.
Bessa et al. [74]	2012	60 patients with T2DM, 20 controls	PDGF-BB can act as a good prognostic marker for the early deterioration of renal function in DN.
Kawano et al. [75]	1993	Diabetic rats and rabbits, non-diabetic rats and rabbits	The rise in aortic SMCs from diabetic rabbits/rats is higher than those in controls, which is linked with the over-expression of PDGF beta-receptors.
Rossi et al. [76]	1998	25 patients with hypertension, 22 controls	The raised level of circulating PDGF might play a significant role in the vascular re-build linked with hypertensive disease.
Wang et al. [77]	2021	30 C57BL/6 mice	Regular aerobic exercise activates the levels of cardioprotective factors like PDGF-BB.
Vantler et al. [78]	2010	Neonatal Wistar rats	PDGF-BB has an anti-apoptotic effect on cardiomyocytes.
Rivera et al. [79]	2021	143 pubertal patients with obesity, 33 controls	PDGF-BB is reduced in young people suffering from obesity in comparison to healthy adolescents.

GDM—gestational diabetes mellitus; IDDM—insulin-dependent diabetes mellitus; SMCs—smooth muscle cells, T2DM—type 2 diabetes mellitus.

**Table 20:** Summary of the studies on the role of M-CSF in skin diseases.

Author	Year	Population	Key Observation
The Role of M-CSF in Skin Diseases			
Pellefigues et al. [80]	2021	6- to 12-week-old Basoph8 mice	M-CSF restricted the influx of pro-inflammatory molecules in the atopic dermatitis.
Li et al. [81]	2022	Balb/C and C57BL/6	Stimulation of wound repairment and hair follicle regeneration was due to M-CSF activation of CD11b-positive myeloid cells.

M-CSF—macrophage colony-stimulating factor.

**Table 21:** Summary of the studies on the role of M-CSF in psoriasis.

Author	Year	Population	Key Observation
The Role of M-CSF in Psoriasis			
Fuentelsaz-Romero et al. [82]	2021	Persistent UA n = 16, Established RA n = 12, PsA n = 10, healthy controls n = 6	M-CSF-dependent anti-inflammatory CD209^+^ macrophages were more enriched in ST from PsA and healthy patients.
Jadon et al. [83]	2017	200 patients with PsC, 127 with pPsA, 117 with PsSpA, 157 with AS without psoriasis, 50 healthy controls	M-CSF can be an indicator of PsA.
Cubillos et al. [84]	2016	21 patients with PsV (within this group, 12 patients with PsA), 15 healthy patients	PBMCs, which are boosted by M-CSF in patients suffering from psoriatic arthritis, are known for the production of a greater amount of pro-inflammatory cytokines.

UA—undifferentiated arthritis, RA—rheumatoid arthritis, PsA—psoriatic arthritis, ST—synovial tissue; PsC—psoriasis without arthritis, pPsA—PsA without axial arthritis, PsSpA—psoriatic spondyloarthritis, AS—ankylosing spondylitis, PBMCs—peripheral blood mononuclear cells.

**Table 22:** Summary of the studies on the role of M-CSF in metabolic syndrome.

Author	Year	Population	Key Observation
The Role of M-CSF in Metabolic Syndrome			
Liu et al. [85]	2009	Adult male Sprague–Dawley Rats	M-CSF is increased in subjects suffering from PDR.
Ko et al. [86]	2007	Homozygous osteopetrotic (Op/Op) mice	Lack of M-CSF in mice caused milder vascular re-modeling, endothelial dysfunction, and oxidative stress.
Radaeva et al. [87]	2019	60 patients with stage II EH	Stage II EH patients with a 10–14 year history of the disease have elevated amounts of serum M-CSF.
Chung et al. [88]	2015	FoxO1^−/−^ mice, FoxO1^fl/fl^ mice, LysM Cre mice, Diabetic db/db mice, nondiabetic db/+ controls	M2-like macrophages lead to inflammation through alleviation of the contribution of FoxO1 and boosting IL-10 expression, which is responsible for hyperglycemia.
Sugita et al. [89]	2007	KKAy (Ay /+) mice, ob/ob mice, C57BL6 mice, op/+ mice	There is no significant alteration of the M-CSF amount in the fatty tissue of obese mice.
Utsunomiya et al. [90]	1995	Male Wistar rats	M-CSF treatment has no influence on serum total cholesterol amount in both hypercholesterolemic and diabetic rats.
Shimano et al. [91]	1990	Four male WHHL rabbits	The total level of cholesterol was reduced in the plasma of rabbits treated with M-CSF.
Inoue et al. [92]	1992	Male WHHL rabbits	M-CSF can avert the progression of atherosclerosis in WHHL rabbits.
Donnelly et al. [93]	1997	10 adult male WHHL rabbits	Rabbits were treated with rhM-CSF raised M-CSF staining, which is associated with declined cholesterol levels in foam cells.
Watanabe et al. [94]	1997	6 male and 6 female 8-month-old WHHL rabbits	M-CSF can influence VSMC function and reduce atherosclerosis in these rabbits.

PDR—proliferative diabetic retinopathy, EH—essential hypertension, FoxO1—forkhead box protein O1, WHHL—Watanabe heritable hyperlipidemic, rhM-CSF—recombinant human M-CSF

**Table 23:** Summary of the studies on the role of VEGF in the skin.

Author	Year	Population	Key Observation
The Role of VEGF in the Skin			
Leung et al. [95]	1989	Bovine pituitary follicular and folliculostellate cells	VEGF is a potent signaling molecule that plays a crucial role in angiogenesis.
Yano et al. [96]	2001	8-week-old female C57BL/6 mice	Perifollicular angiogenesis is regulated by VEGF.

VEGFRs—VEGF receptors.

**Table 24:** Summary of the studies on the role of VEGF in skin diseases.

Author	Year	Population	Key Observation
The Role of VEGF in Skin Diseases			
Choi et al. [97]	2003	N1-48 patients with SSc, N2-30 control patients	The severity of nailfold capillary loss is positively correlated with VEGF level.
Tedeschi et al. [98]	2009	N1-83 patients with CU, N2-53 healthy patients	VEGF level is correlated with CU severity.

SSc—systemic sclerosis, CU—chronic urticaria.

**Table 25:** Summary of the studies on the role of VEGF in psoriasis.

Author	Year	Population	Key Observation
The Role of VEGF in Psoriasis			
Young et al. [99]	2006	N1-292 psoriatic patients, N2-101 control patients	Elevated levels of VEGF were found in psoriatic plaques.
Akman et al. [100]	2009	1 psoriatic patient	Psoriasis complete remission was noted after and during treatment with bevacizumab, a monoclonal antibody that inhibits VEGF.

AP-1—activator protein-1, KCs—keratinocytes.

**Table 26:** Summary of the studies on the role of VEGF in metabolic syndrome.

Author	Year	Population	Key Observation
The Role of VEGF in Metabolic Syndrome			
Blann et al. [101]	2001	20 patients with hyperlipidemia	Plasma VEGF levels were elevated in patients with hyperlipidemia, and these levels were reduced with lipid-lowering therapy.
Facemire et al. [102]	2009	Male 129S6/SvEv mice	VEGF signaling was found to be important for maintaining normal blood pressure.

NOS—nitric oxide synthase, NO—nitric oxide.

**Table 27:** Summary of the studies on the role of TGF-α in the skin.

Author	Year	Population	Key Observation
The Role of TGF-α in the Skin			
Grellner et al. [103]	2005	74 TGF-α skin wounds	TGF-α stimulated angiogenesis and the formation of new blood vessels.

TGF-α—transforming growth factor alpha.

**Table 28:** Summary of the studies on the role of TGF-α in skin diseases.

Author	Year	Population	Key Observation
The Role of TGF-α in Skin Diseases			
Koyama et al. [104]	1997	1 patient with skin symptoms	High levels of serum TGF-α over a long time were a major cause of acanthosis nigricans.
Partridge et al. [105]	1989	Samples of normal skin and oral SCC	The production of TGF-α was increased in oral SCC cells.

EGFR—epidermal growth factor receptor.

**Table 29:** Summary of the studies on the role of TGF-α in psoriasis.

Author	Year	Population	Key Observation
The Role of TGF-α in Psoriasis			
Elder et al. [106]	1989	Skin samples from the normal epidermis uninvolved epidermis and lesional epidermis from psoriatic patients	TGF-α mRNA levels were significantly higher in lesional psoriatic skin.
Higashiyama et al. [107]	1991	N1-4 psoriatic patients, N2-6 healthy patients	TGF-α is involved in the induction or the maintenance of hyperproliferation of psoriatic epidermal keratinocytes.

**Table 30:** Summary of the studies on the role of TGF-β in the skin.

Author	Year	Population	Key Observation
The Role of TGF-β in the Skin			
Hirai et al. [108]	2019	Itgb6^−/−^ Itgb8DKC mice	TGF-β plays a role in the maintenance of both TRM cells and CMT cells in the skin.
Schmid et al. [109]	1993	Skin biopsies from chronic non-healing decubitus ulcers	TGF-β3 plays a central role in epidermal maintenance.

TRM cells—epidermal-resident memory T cells, CMT cells—circulating memory T cells, VV—vaccinia virus, TGF-β3—transforming growth factor beta 3, TGF-β1—transforming growth factor-beta 1, TGF-β2—transforming growth factor beta 2.

**Table 31:** Summary of the studies on the role of TGF-β in skin diseases.

Author	Year	Population	Key Observation
The Role of TGF-β in Skin Diseases			
Santiago et al. [110]	2005	C3H mice	Topical application of P144, a peptide inhibitor of TGF-β1, significantly reduced skin fibrosis.
Denton et al. [111]	2007	45 patients with SSc	CAT-192, an anti-TGFβ1 drug, showed no evidence of efficacy in the treatment of SSc in doses up to 10 mg/kg.

**Table 32:** Summary of the studies on the role of TGF-β in psoriasis.

Author	Year	Population	Key Observation
The Role of TGF-β in Psoriasis			
Wataya-Kaneda et al. [112]	1996	Six psoriatic skin samples, normal human skin	The decrease of TGF-β2 may be involved in the excessive proliferation of keratinocytes, which is a hallmark of psoriasis.
Flisiak et al. [113]	2002	41 patients with chronic plaque-type psoriasis	TGF-β1 may be a biomarker of psoriasis activity.

**Table 33:** Summary of the studies on the role of TGF-β in metabolic syndrome.

Author	Year	Population	Key Observation
The Role of TGF-β in Metabolic Syndrome			
Lin et al. [114]	2017	N1-533 patients with MS, N2-2467 patients without MS	TGFBR2 genes may be associated with MS.
Herder et al. [115]	2009	N1-460 patients with T2DM, N2-1474 patients without T2DM	Elevated serum concentrations of the TGF-β1 indicate an increased risk for T2DM.

TGFBR2—transforming growth factor beta receptor 2, SNPs—single-nucleotide polymorphisms.

**Table 34:** Summary of the studies on the role of EGF in the skin.

Author	Year	Population	Key Observation
The Role of EGF in the Skin			
Cohen et al. [116]	1962	Submaxillary glands isolated from adult male Swiss Webster mice	EGF leads to cell growth, proliferation, differentiation, and survival.
Bhora et al. [117]	1995	Fetal bovine serum, human skin	EGF is a particularly potent stimulator of epithelialization.

EGF—epidermal growth factor, EGFR—epidermal growth factor receptor, FGF—fibroblast growth factor, IGF-1—insulin-like growth factor-1.

**Table 35:** Summary of the studies on the role of EGF in skin diseases.

Author	Year	Population	Key Observation
The Role of EGF in Skin Diseases			
Choi et al. [118]	2018	Six-week-old male NC/Nga mice	EGF’s anti-inflammatory and antimicrobial properties make it a promising candidate for the treatment of a chronic skin condition.
Paik et al. [119]	2013	C57BL/6 mice	EGF treatment favored primary hair recovery through the dystrophic anagen pathway after CIA.

AMPs—antimicrobial peptides, HEKs—human epidermal keratinocytes, HKSA—heat-inactivated *S. aureus* (HKSA), TSLP—thymic stromal lymphopoietin, CIA—cyclophosphamide-induced alopecia.

**Table 36:** Summary of the studies on the role of EGF in psoriasis.

Author	Year	Population	Key Observation
The Role of EGF in Psoriasis			
Flisiak et al. [120]	2014	51 patients with plaque psoriasis	The positive correlation between EGF levels and PASI suggests that EGF may be a marker of disease severity
Nanney et al. [121]	1986	Psoriatic skin, healthy skin	The retention of EGF receptors may reflect the abnormal and incomplete differentiation in active psoriatic lesions.

sEGFR—soluble epidermal growth factor receptor.

**Table 37:** Summary of the studies on the role of EGF in metabolic syndrome.

Author	Year	Population	Key Observation
The Role of EGF in Metabolic Syndrome			
Kyohara et al. [122]	2020	N1-106 patients with T2DM, N2-47 patients without T2DM	Soluble EGFR may play a role in the development of hepatic insulin resistance.
Belmadani et al. [123]	2008	Diabetic db/db and nondiabetic (control) mice	An EGFR inhibitor, AG1478, suggested that EGFR is a potential target for overcoming diabetic small artery complications.

MRAs—mesenteric resistance arteries.

**Table 38:** Summary of the studies on the role of IGF in the skin.

Author	Year	Population	Key Observation
The Role of IGF in the Skin			
Tavakkol et al. [124]	1992	Human skin biopsies	IGF-1 directly stimulates keratinocyte proliferation.
Lewis et al. [125]	2009	Human skin biopsies	The age-related decline in IGF-1 production may be due to senescence of dermal fibroblasts.

IGF-1—insulin-like growth factor 1.

**Table 39:** Summary of the studies on the role of IGF in skin diseases.

Author	Year	Population	Key Observation
The Role of IGF in Skin Diseases			
Rahaman et al. [126]	2016	N1-80 patients with acne vulgaris, N2-80 healthy patients	IGF-1 may play a role in the development of acne.
Tan et al. [127]	2016	N1-25 patients with oral lichen planus, N2-13 healthy patients	IGF-1 may stimulate the proliferation of T cells, which may contribute to the pathogenesis of OLP.

**Table 40:** Summary of the studies on the role of IGF in psoriasis.

Author	Year	Population	Key Observation
The Role of IGF in Psoriasis			
Miura et al. [128]	2000	N1-9 psoriatic patients, N2-7 healthy patients	Dermal fibroblasts may contribute to the epidermal hyperplasia of psoriasis by promoting keratinocyte proliferation through IGF-1.
El-Komy et al. [129]	2011	N1-24 psoriatic patients, N2-12healthy patients	Down-regulation of IGF-1 following methotrexate or PUVA treatment may be due to a decrease in proinflammatory cytokines, inflammatory cellular infiltration, or an effect on local fibroblast activity and proliferation.

CF—control fibroblasts.

**Table 41:** Summary of the studies on the role of IGF in metabolic syndrome.

Author	Year	Population	Key Observation
The Role of IGF in Metabolic Syndrome			
Saydah et al. [130]	2009	5903 participants	Each component of MS was associated with lower levels of IGF-1, IGF-BP3, and the IGF-I/IGF-BP3 ratio.
Efrastadias et al. [131]	2006	N1-123 patients with MS, N2-47 patients without MS	Low IGF-1 and high CRP levels were associated with an increased number of MS components.

IGF-BP3—IGF-binding protein 3, CRP—C-reactive protein.

**Table 42:** Summary of the studies on the role of TNF inhibitors and BMI.

Author	Year	Population	Key Observation
The Roles of TNF Inhibitors in BMI			
Tan et al. [132]	2013	143 psoriatic patients	Therapy with adalimumab and infliximab is associated with a significant increase in body weight and BMI.
Hojgaard et al. [133]	2016	943 PsA patients	Obesity was associated with higher disease activity and seemed to diminish response and adherence to TNFIs in PsA.
Naldi et al. [134]	2008	8072 psoriatic patients	BMI affects the early clinical response to systemic treatment for psoriasis.

TNFI—tumor necrosis factor inhibitors, PsA—psoriatic arthritis.

**Table 43:** Summary of the studies on the role of IL-17A inhibitors and their influence on BMI.

Author	Year	Population	Key Observation
The Role of IL-17A Inhibitors in BMI			
Takamura et al. [135]	2018	N1-68 psoriatic patients, N2-18 patients treated with infliximab, N3-30 psoriatic patients treated with Ustekinumab, N4-20 patients treated with secukinumab	Infliximab increases body weight in the psoriatic patients, whereas ustekinumab and secukinumab do not affect the body weight in these patients.
Egeberg et al. [136]	2018	Psoriatic patients	Ixekizumab was not associated with any significant changes in body weight.
Reich et al. [137]	2017	3855 psoriatic patients	Ixekizumab is an effective and safe treatment option for patients with psoriasis, regardless of their body weight.
Piros et al. [138]	2021	35 psoriatic patients	Anti-interleukin-17 therapy in severe psoriatic patients does not cause significant changes in body composition parameters.

**Table 44:** Summary of the studies on the role of IL-12/23 Inhibitors and their influence on BMI.

Author	Year	Population	Key Observation
The Role of IL-12/23 Inhibitors in BMI			
Gisondi et al. [139]	2013	N1-79 psoriatic patients treated with Ustekinumab, N2-83 psoriatic patients with infliximab	Ustekinumab does not increase BMI in patients with chronic plaque psoriasis.
Lebwohl et al. [140]	2010	N1-1331 psoriatic patients treated with Ustekinumab, N2-665 psoriatic patients treated with placebo	Patients with a BMI greater than 25 kg/m² had a lower response rate to ustekinumab compared to patients with a lower BMI.
Papp et al. [141]	2008	N1-1230 psoriatic patients, N2-820 psoriatic patients treated with ustekinumab, N3-410 psoriatic patients with placebo	Obesity may be a factor that influences the effectiveness of ustekinumab in treating psoriasis.
Young et al. [142]	2011	1175 psoriatic patients	Ustekinumab may be more effective in patients with high BMI in comparison to etanercept.
Zweegers et al. [143]	2016	N1-186 psoriatic patients treated with adalimumab, N2-238 psoriatic patients treated with etanercept, N3-102 psoriatic patients treated with ustekinumab	Higher BMI is a predictor for discontinuation due to ineffectiveness in etanercept and ustekinumab.
